# Long read RNA sequencing of transposable elements from single cells using CELLO-seq

**DOI:** 10.1038/s41596-025-01203-2

**Published:** 2025-07-16

**Authors:** Sophie A Marlow, Lauryn A Deaville, Rebecca V Berrens

**Affiliations:** 1 Department of Biochemistry, Oxford University, South Parks Rd, Oxford OX1 3QU; 2 MRC Weatherall Institute of Molecular Medicine, Oxford University, John Radcliffe Hospital, Oxford OX3 9DS

## Abstract

Transposable elements (TEs) form 50% of the mammalian genome sequence and their expression contributes to processes from development to disease. Due to the abundance and high sequence similarity of TEs, short-read single-cell sequencing methods promote ambiguous mapping to many almost identical TE loci across the genome. To enable mapping of TE-derived reads to unique genomic loci, we have developed a protocol for single-cell long-read RNA sequencing (CELLO-seq). CELLO-seq enables error correction of long reads through incorporation of long 50 nucleotide unique molecular identifiers (UMIs) and high PCR duplicate numbers. Taken together, these features of CELLO-seq enable high-fidelity mapping of expression data to highly sequence-similar young TEs, as well as to gene and TE isoforms. This will enable in depth exploration into the interaction of individual TEs and genes within single cells. This protocol is designed to be accessible for users with experience in molecular biology and transcriptomic analysis, and can be completed within a week from cell isolation through to quantification.

## Introduction

Transposable elements (TEs) are estimated to contribute to 25% of human *cis*-regulatory elements^1–4^. Examples of TE-derived *cis*-regulatory activity are emerging across biology, including in neuronal differentiation and early development^5–7^. Here, the stage-specific TE expression has potential to contribute to cell fate decisions^8–10^. Despite their extensive biological roles, TE are often excluded from transcriptomic and genomic studies due to the abundance and high similarity of their sequences^11^. TEs produce multiple sequence-similar copies via their transposition throughout the genome over the course of evolution^12^. As new copies become domesticated within the host genome they begin to diverge from the consensus, with the increase in polymorphisms enabling differentiation of loci within a particular TE family. However, for the evolutionarily youngest TE families that remain retrotransposition-active, such as L1HS, SVA and Alu in humans and L1Md, SINE-B1, MERVL, and IAP elements in mice, many genomic copies remain almost identical^13–16^. This is an important consideration when detecting TE expression using RNA sequencing. Here, mapping individual TE transcripts to their locus of origin is a challenge^17^.

Short reads, typically up to 200 nucleotides (nt), often cannot be accurately mapped to genomic regions that originate from transposable elements because the reads lack sufficient polymorphisms to distinguish between similar sequences.^18,19^. Several computational frameworks have been developed to statistically assign reads to transposable element loci or their higher-order subfamilies on bulk^18,20,21^ and single-cell^22,23^ scales, respectively. However, computational methods often struggle to detect evolutionarily young transposable elements. These exhibit the greatest sequence similarity due to the lack of divergence between loci^20,24^. Consequently, young transposable elements remain under-represented in transposable element studies to date and are often only studied at the level of families of similar sequences^3^. This leaves many remaining questions surrounding the roles of individual TEs, which is critical to our understanding of their *cis*-regulatory potential. Despite this, young TEs represent the small proportion of elements that remain transpositionally active, making them of particular interest to study in the context of both health and disease^13,15,16,25,26^.

### Development of the protocol

**Figure 1:**
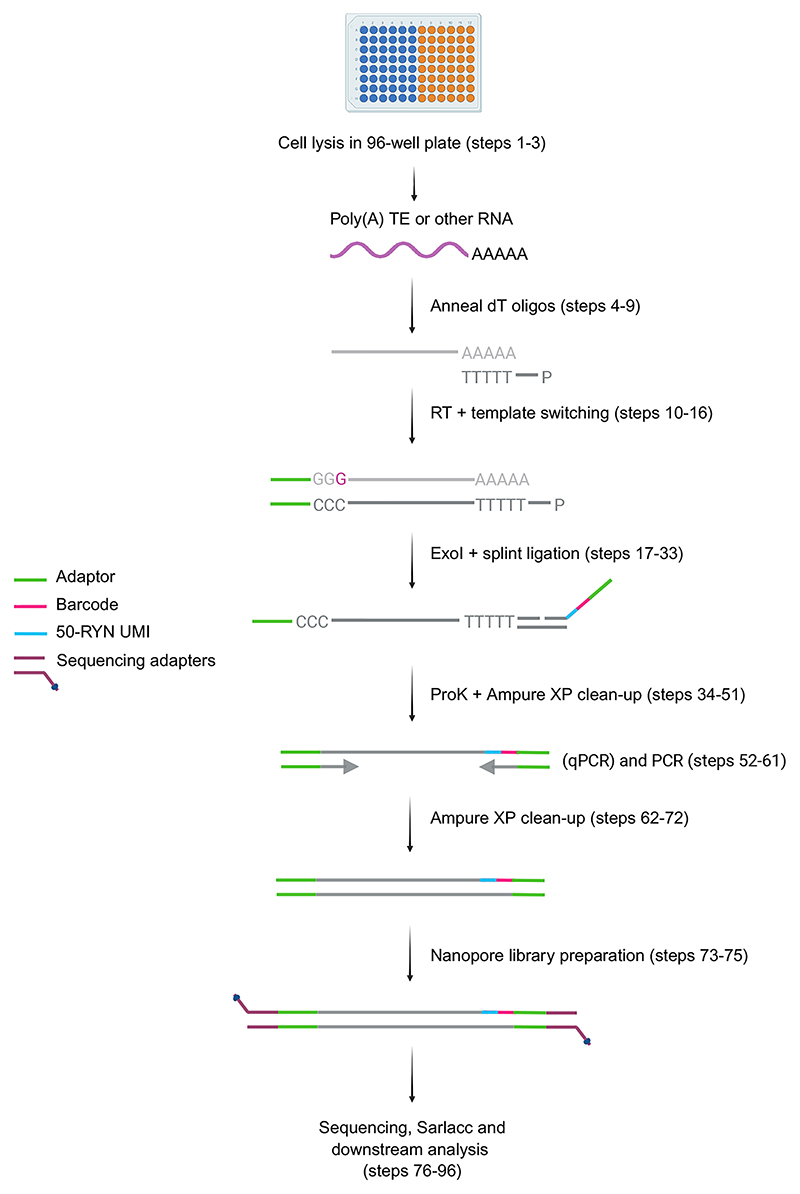
Schematic representation of the CELLO-seq wet lab workflow labelled with key steps in the protocol. Single cells for CELLO-seq are first sorted into individual wells of a 96 well plate containing lysis buffer. dT oligos are annealed to the poly(A) tail of RNAs, priming reverse transcription and template switching. Following ExoI treatment, splint oligos are ligated, consisting of an adaptor, barcode and 50-RYN UMI (green, pink and blue, respectively). Proteinase K digestion of contaminant proteins is followed by an AMPure XP clean-up and PCR. After additional AMPure XP clean-up steps, ONT library preparation can be performed, whereby ONT sequencing adaptors (purple) are ligated onto the amplified cDNAs. The ONT library is sequenced on a MinION or PromethION flow cell and the fastq files are analysed with the sarlacc pipeline. Abbreviations: ProK (Proteinase K), ExoI (Exonuclease I), UMI (unique molecular identifier). Figure created with BioRender.com.

Here we share our detailed protocol for our recently published method, CELLO-seq^19^ (Figure 1). CELLO-seq enables single-cell long-read RNA sequencing of TEs at the locus level, through a combination of long reads, 50 nt long unique molecular identifiers, and a high number of PCR duplicates to allow for error correction of reads^27–29^. Together, these features of the CELLO-seq protocol enable detection of TE expression at the single-cell level. Through this highly accurate unique molecular identification, CELLO-seq has far reaching applications even beyond the transposon field and can be used for both gene and TE isoform detection, as well as retroviral sequencing of infected cells. Further, CELLO-seq can be applied to any cell type, including cryopreserved cells and fresh tissue samples.

**Extended Data Figure 1:**
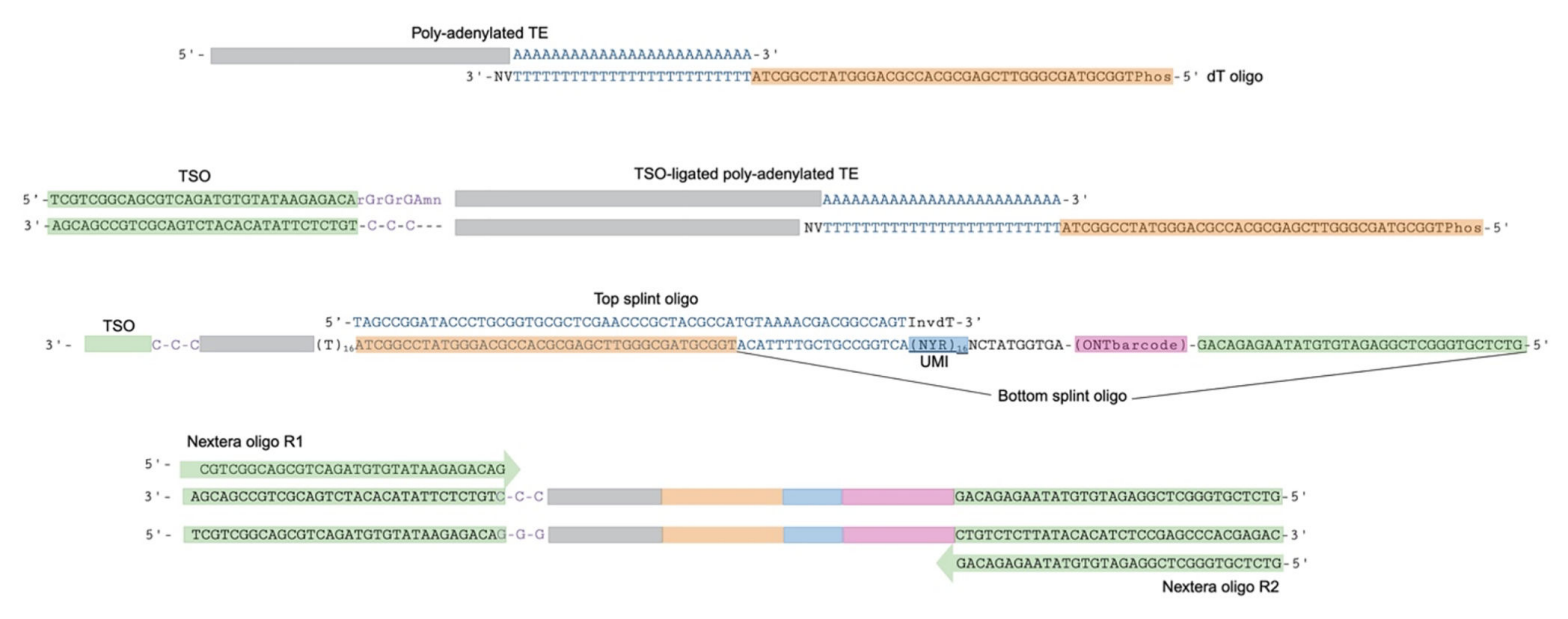
Oligo interactions in CELLO-seq. Schematic depicting the interactions between oligos used in CELLO-seq. A phosphorylated dT oligo is annealed at the poly(A) tail of polyadenylated TE transcripts, priming reverse transcription. TSO is ligated to the 5’ end of the poly(A) TE. Annealed top and bottom splint oligos are annealed and ligated to the 5’ end of the reverse transcribed transcripts. Annealed splint oligos contain a UMI, ONT barcode and an annealing site for Nextera oligo R1 at the 5’ end. At the 3’ end of the cDNA is the priming site for Nextera oligo R2. Figure created with BioRender.com.

Fundamental to the accurate assignment of TEs to their unique loci is the splint oligo design (Extended Data Figure 1). The splint oligos consist of a unique molecular identifier (UMI) and a ONT barcode to label unique mRNAs from individual cells. UMIs allow identification of PCR duplicates which can be used to generate consensus sequences for each full-length TE transcript, either through de-duplication or error correction.

### Unique molecular identifier design

The RYN UMI design helps to overcome the innate disadvantage of ONT sequencing in calling homopolymer sequences. CELLO-seq UMIs are a repetition of purine (R), pyrimidines (Y), and any nucleoside (N), to prevent the generation of homopolymers. Homopolymers of any length still present a challenge to ONT sequencing^31^, but the unique design of CELLO-seq UMIs circumvents this. Furthermore, constant regions between the ONT barcode and the UMI improves UMI identification. Preventing the misassignment of barcode nucleotides as those belonging to UMIs enables the UMI sequence to be clearly distinguished from the barcode^32^.

**Figure 2:**
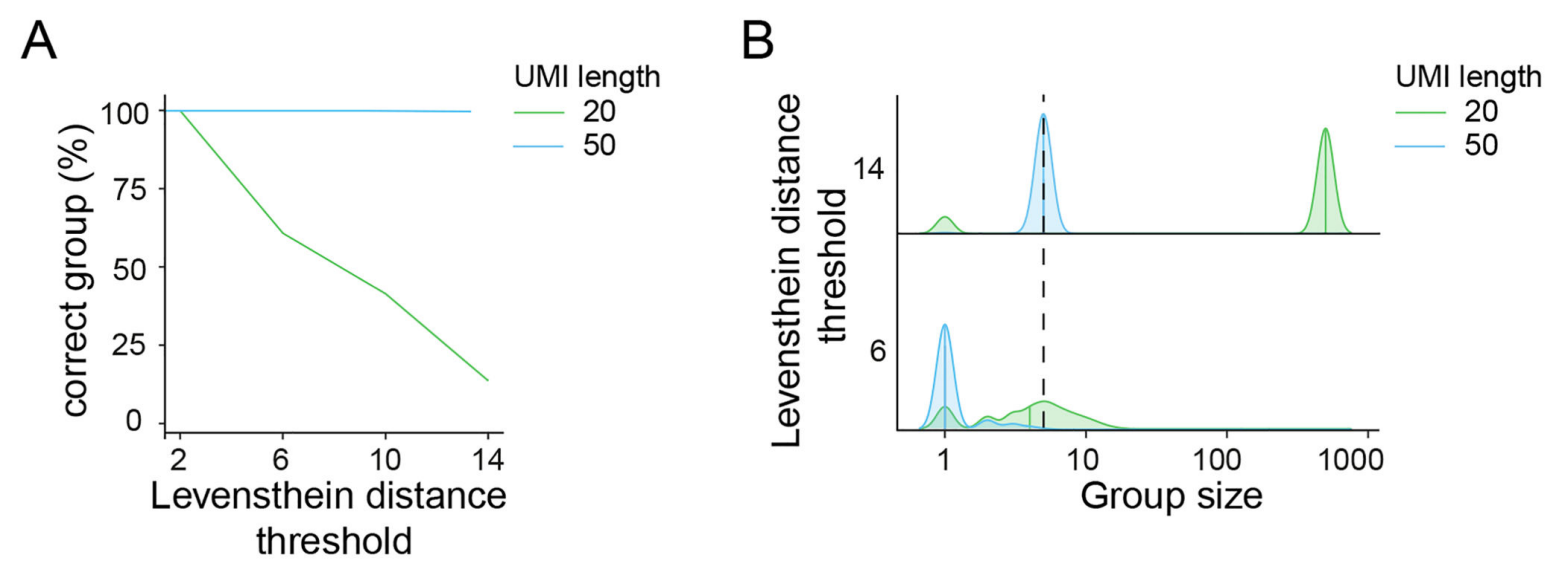
Schematic of error correction with 20 or 50 nt UMI. (A) UMI simulations for the proportion of TEs assigned to the correct group after grouping (y-axis) against Levenshtein distance thresholds (x-axis). (B) Distribution plot of group sizes against their Levenshtein distance threshold after correct grouping for each UMI length of the simulated reads. Dotted line represents the known group size as the simulation was performed assuming 5 PCR replicates of each read, whilst avoiding over grouping, for high and low Levenshtein threshold. Figure adapted with permission from ref.^19^ Springer Nature.

CELLO-seq UMIs are 50 nt long, approximately 5 times longer than in other sequencing methods and necessary to perform accurate error correction and mapping of individual transcripts to unique transposable element loci (Figure 2) ^33,34^. To computationally assign the UMI to each transcript, Levenshtein distance thresholds are used. Levenshtein distances represent a measure of the number of single-nucleotide edits required to change one sequence into another^30^. Due to the high error rate of ONT sequencing, it is important to find the correct balance between accurately grouping reads together and avoiding overly strict grouping, which could cause each read to be placed in a group alone. Overall, longer UMIs lead to more accurate read groupings by reducing the probability of different cDNA molecules being assigned the same UMI (Figure 2A). Previous UMI simulations revealed that for UMIs of 20 nt length, a stringent Levenshtein threshold of 6 was necessary to group the majority of reads accurately^19^. However, while most reads were correctly grouped, many reads were being grouped too strictly into individual read groups, preventing error correction (Figure 2B). The same UMI simulations, revealed that increasing the UMI length to 50 nt enables increasing of the Levenshtein threshold to 14, decreasing the stringency; this simultaneously increases the number of reads grouped together without risking a loss in grouping accuracy (Figure 2B). With this rationale, the 50 nucleotide (nt) UMIs used in this protocol have replaced the 22 nt UMIs used previously in CELLO-seq^19^.

### Blocked template switch Oligo

Template switch oligos (TSOs) are used to transcribe full-length mRNAs^35^. In CELLO-seq, unlike the LNA-based TSO used in SMART-seq2, an RNA-based TSO is used to provide higher cDNA output. A blocked TSOs with an amine group at the 3’-end is inhibited from acting as a primer but still provides template switch activity. This prevents TSOs acting as primers for breathing amplification in second strand cDNA generation during reverse transcription^36^.

### Strengths of the protocol

**Extended Data Figure 2:**
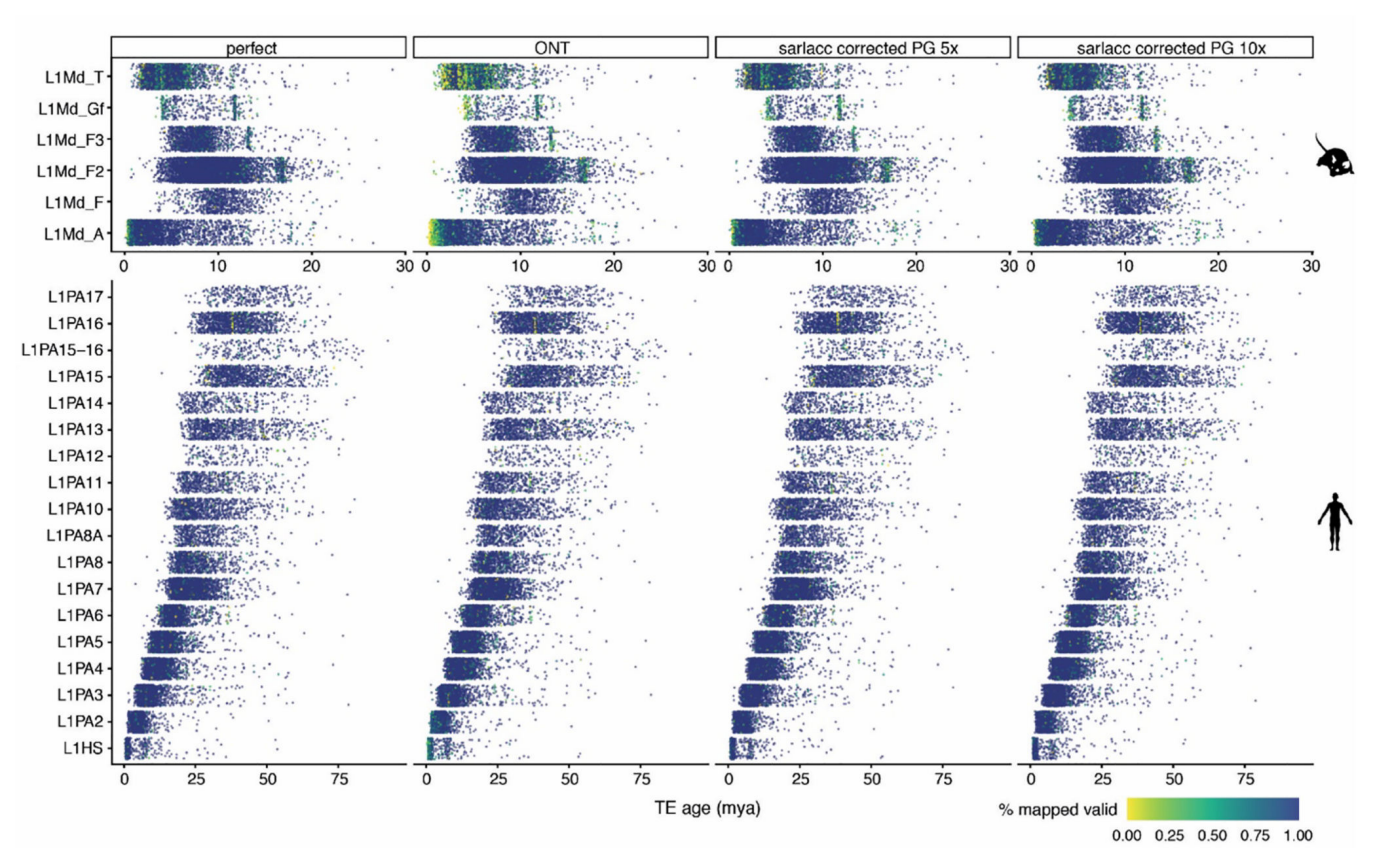
Mappability of TEs of across ages and different sequencing approaches. Jitter plot of TE subfamily (y-axis) by TE age (million years ago) for different simulation types. The % of mapped reads is represented by yellow being 0% mapped and dark blue being 100% mapped. Mouse L1 top panel and human L1 bottom panel. Simulation type: perfect = perfect read identity, ONT = Read identity achievable with ONT, ONT 5x = ONT read identity with 5x coverage, sarlacc corrected 5x = ONT read identity score with 5x coverage combined with sarlacc error correction, sarlacc corrected 10x = ONT read identity score with 10x coverage with sarlacc error correction. PG = perfect grouping. ONT error rate was simulated using Badread and for simulating a perfect read identity, Illumina reads were used as a baseline. The ability to map simulated reads to their valid loci was assessed by processing ONT simulated reads with 5X and 10X coverage using the sarlacc pipeline to produce error corrected reads. For the purposes of simulation, simulated reads were aligned to repeat sequences of young L1PA and L1Md elements in the alignment stage. Perfect simulated, ONT simulated and sarlacc processed reads were aligned against the reference genome using minimap2 and the location of aligned reads were evaluated against their true location. Original figure from^19^.

**Extended Data Figure 3:**
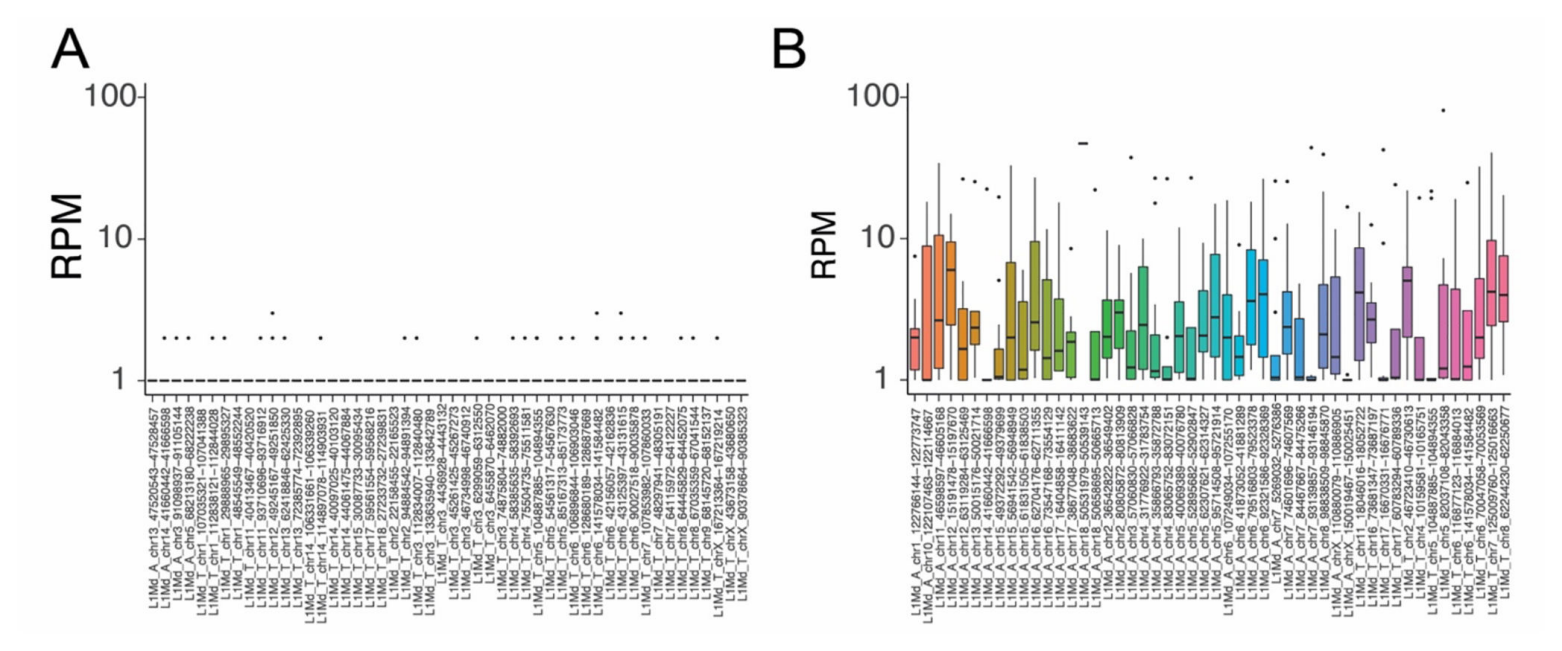
Mouse Young L1 expression in ScNaUmi-seq data versus CELLO-seq data. Boxplots of the 50 most highly expressed young L1 unique loci on the x-axis, against their log10 counts on the y-axis for illumina ScNaUmi-seq data (A) versus CELLO-seq data (B) of mouse embryonic stem cells cultured in 2i medium. For the boxplots, the median, first and third quartiles are a box and the whiskers indicate the most extreme data point within 1.5 lengths of the box. Original figure from^19^.

**Extended Data Figure 4:**
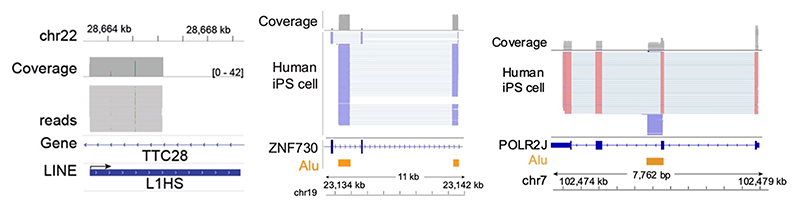
CELLO-seq provides reads beginning at the transcription start site of TEs. Example genome browser tracks for human genome depicting (left) a L1HS, with the direction of transcription shown via the arrow. Reads covering the L1HS element are shown, which begin at the transcription start site of the TE. Reads overlapping an Alu element (middle) in the human genome that has been spliced into the ZNF730 gene and serves as an alternative promoter, versus an Alu element (right) which has not been spliced into the genome, transcribed by its own promoter. Original figure from^19^.

CELLO-seq offers several advantages over other methods for analysing TE expression. Notably, CELLO-seq allows for the precise mapping of young TE transcripts to their loci of origin (Extended Data Figure 2), afforded by several unique features as outlined below:

- CELLO-seq involves a qPCR quality control step to optimise library complexity. Performing qPCR for every new cell type used in CELLO-seq ensures over-amplification of cDNA is avoided, preventing over-representation of specific amplified cDNAs.
- The adapters used in CELLO-seq have been carefully designed to include a constant region which interrupts the barcode and UMI sequences, improving UMI recognition and results in fewer reads being discarded when compared to other methods^32^.
- Unlike other long-read sequencing methods, the UMIs used in CELLO-seq have an increased length of 50 nt. Long UMIs enable assignment of reads to correct groups, alongside subsequent error correction and high-accuracy mapping of TE transcripts.
- The RYN design of CELLO-seq UMIs avoids errors associated with basecalling of homopolymer sequences with error-prone ONT sequencing.
- Long reads obtained using CELLO-seq improve mapping accuracy of reads derived from the youngest transposable elements over short read methods (Extended Data Figure 3).
- Strand switching at the 5’ capped sequence of cDNAs optimises mappability of young TEs by increasing abundance of reads over the promoter region of TEs, which is the source of greatest heterogeneity amongst transcripts.
- Priming at the 3’ poly(A) tail and strand switching at the 5’ capped sequence allows for the full TE to be sequenced, allowing for TE chimeric transcripts to be detected, alongside gene and TE expression.
- It is possible to distinguish between active and readthrough transcription of TEs with CELLO-seq through identification of transcription start and end sites (Extended Data Figure 4).

### Overview of the procedure

CELLO-seq is a plate-based sequencing method (Figure 1). Before starting CELLO-seq, single cells must first be sorted via flow activated cell sorting (FACS) into individual wells of a 96-well plate containing lysis buffer (Steps 1-3; Figure 1). Contamination and RNA degradation must be limited as much as possible by ensuring FACS sorting takes place in sterile conditions and at 4°C, respectively. Additionally, a ribonuclease inhibitor is included in the lysis buffer to limit RNA degradation. Cell lysates in 96-well plates can be either processed immediately or stored at -80°C until later processing.

The CELLO-seq protocol begins with annealing dT oligos onto 3’ polyadenylated RNA sequences which act as a primer for subsequent reverse transcription (RT) (Steps 4-9). In addition, some short interspersed nuclear elements (SINEs), such as mouse B1/B2 or human Alu elements, contain internal poly(A) sequences. These can serve as oligo-dT primer binding sites and enable transcripts to be also detected with CELLO-seq. Reverse transcription and template switching is performed at 53°C for 10 min followed by 42°C for 2 hours (Steps 10-16). Secondary structure formation more frequently takes place with TE RNA compared to other RNA transcripts due to their highly repetitive sequences^37,38^. Therefore, to limit secondary structure formation and RT stalling, we use an initial 53°C heating at the start of the RT reaction. The single stranded binding protein, ET SSB, binds to full-length RNA molecules and is included in the RT reaction to increase the length of cDNA transcript outputs for long-read sequencing. Any remaining dT oligo and TSO from template switching must first be digested with Exonuclease I (ExoI) (Steps 17-22). ExoI digestion prevents additional ligation of dT or TSO onto cDNA. After ExoI treatment, splint oligos are ligated onto each cDNA (Steps 23-33). Once splint oligos are ligated, proteins present in the buffers required for exonuclease I digestion and RT buffers are digested with proteinase K (Steps 34-38) to avoid disruption of subsequent DNA purification steps with AMPure XP beads (Steps 39-51). A fraction of the library is used for quantitative PCR (qPCR) with Evagreen to determine the number of PCR cycles required for library preparation (Step 52-56). Evagreen is a non-fluorescent dye that becomes fluorescent upon binding to dsDNA. For a qPCR with 1/16th of the original cDNA volume, the number of PCR cycles can be calculated by taking the number of cycles before exponential growth and subtracting 4, given the 16x higher input amount for the final PCR (Steps 57-61). This ensures sufficient material for sequencing while avoiding overamplification and excessive PCR duplicates. Typically, the number of cycles ranges between 15 and 29, depending on the cell type used. This high number of PCR cycles when compared to other single-cell sequencing protocols e.g. SMART-seq2^29,33^, enables for the high number of PCR duplicates to be achieved for error correction to take place.

After PCR, two sequential DNA purifications with 0.5x AMPure XP beads enable removal of shorter DNA fragments from the library, including those derived from splint oligo contaminants and primer dimers (Steps 62-72). The mean cDNA length and concentration are measured for the completed CELLO-seq library, which also provides an indication of the quality (Steps 73 and 74). Fragments of ~2 kb typically indicate full-length cDNAs suitable for Oxford Nanopore Technologies (ONT) library preparation and sequencing (step 75). After sequencing, the fastq files retrieved are then subject to de-duplication or error correction of reads in the Sarlacc computational pipeline (Steps 76-96). Following this analysis pipeline, it is possible to identify TE expression from individual loci across a range of TE families.

### Comparison with other methods

#### Comparison with computational methods for measuring TE expression

Current transcriptomic approaches used to study TEs range in the resolution they can achieve^39^. Bulk RNA-sequencing has effectively been used to decipher family level^21,40,41^ as well as locus specific expression^42–46^, while single-cell methods remain limited to detecting the expression of TE sub-families or families rather than loci^23^. Locus-specific single-cell computational methods give both false positive and false negative TE expression due to incorrect allocation of reads^22^. Single-cell compatible methods also exist to estimate the expression of specific TE families, such as LINE1^47–49^ or group similar cells together to assign TE expression^50^. These methods also often fail to distinguish genic reads from transposable element derived transcripts. CELLO-seq is the only method, to the best of our knowledge, that combines single-cell sequencing of the transcriptome with the ability to map locus-specific expression of TEs across all TE sub-families.

#### Comparison with other single-cell long-read RNA-seq methods

One of the key differences between CELLO-seq and other single-cell long-read methods is the use of blocked TSOs. Sequencing methods making use of unblocked TSOs may be susceptible to breathing amplification at low temperatures^36,51^. During the reverse transcription (RT) step, the DNA duplex structure can become accessible to primers and polymerases, allowing for amplification and potentially resulting in an overestimation of expressed transcripts. TSO blocking prevents breathing amplification by stopping TSOs from acting as primers which would otherwise be present on the 3’ and 5’ ends of reads. This is crucial to avoid overestimation of transcript expression levels using UMIs. Many transcripts in 10X, when combined with long-read sequencing, have TSOs on each of the 5’ and 3’ ends. Some methods include a selection step against these transcripts or filters them out computationally^28^. Other methods, such as MAS-seq avoid TSO artifacts by excluding them from concatenation prior to PacBio sequencing^52^.

Secondly, in comparison to other single-cell long-read methods, CELLO-seq uses much longer UMIs. Extensive analysis of the length of UMIs required to allow for error correction of reads and accurate mapping of long reads to unique young transposable element loci revealed 50 nt UMIs are optimal (Figure 2). Hence, methods such as 10X, PacBio and conventional ONT sequencing that uses 8-12nt UMIs, are not ideal for accurate locus-specific mapping of reads from the youngest TEs. Error correction has been incorporated into other single-cell long-read sequencing approaches based on ONT. These include ScNaUmi-seq^28^, which also employs a UMI-based approach for correcting the ONT error rate. ScNaUmi-seq relies on accurately mapped short reads to enable error correction of long reads, however this method identified low levels of young TE expression compared to CELLO-seq. This is attributed to unreliable mapping of short reads to young TE sequences, meaning the full repertoire of TEs expressed in each cell cannot be explored (Extended Data Figure 3). scCOLOR-seq^53^ also uses a UMI-based approach in combination with ONT sequencing to error correct UMIs and barcodes, with high levels of success. However, once again this technique is not optimised for analysis of TE expression levels, particularly for young TEs. scCOLOR-seq has an emphasis on applying error correction to UMIs and barcodes, to ensure the correct assignment of reads to individual cells. CELLO-seq offers a more extensive error correction approach. Not only is error correction applied to the UMI and barcode identifiers, but to the entire read itself, capturing sequencing errors in the transcript. The capability to error correct entire reads allows for the youngest TEs with minimal sequence deviations between individual TE transcripts to be distinguished.

Rolling Circle Amplification to Concatemeric Consensus (R2C2)^54^ is another ONT-based method which corrects for sequencing errors using a concatemer amplified by rolling circle amplification. Concatenating multiple cDNA molecules together for sequencing improves the error rate of ONT-sequencing by enabling consensus sequences to be generated. However, the circular nature of the reads results in a relatively low raw read throughput. R2C2 does not incorporate UMI sequences into DNA, meaning the opportunity to error correct PCR duplicates of cDNA molecules is lost. Therefore, PCR errors cannot be error corrected with this method. More recently, a method for including UMIs in R2C2 library preparation has been developed to enable enhanced error correction and, in turn, higher levels of sequencing accuracy^55^. However, the sequencing depth achieved by R2C2 remains relatively low, limiting the ability to detect transcripts of lowly expressed gene- and TE-derived isoforms. Furthermore, because R2C2 is based on rolling circles, there is a bias for shorter transcripts to be circularised over longer transcripts, biasing analysis towards shorter cDNAs in general^56^.

MAS-ISO-seq is another concatenation-based approach which uses PacBio sequencing to identify full-length transcripts of isoforms in single cells^52^. Consensus sequences generated through error correction of several circular passes of concatenated reads enables for isoform detection. The input material for MAS-ISO-seq is a cDNA library prepared with a 10x technologies. 10X cDNA libraries result in an average cDNA length of 1.2 kb, while CELLO-seq cDNA libraries have an average length of 2 kb. CELLO-seq library preparation coupled to MAS-ISO-seq has potential to increase the likelihood of capturing expression heterogeneity of highly sequence similar TEs with longer cDNAs. CELLO-seq is compatible with different sequencing technologies. While PacBio has lower throughput than ONT, it can be used in combination with cDNA concatenation (as in MAS-ISO-seq) as an alternative, particularly if PacBio is better suited to specific user needs.

### Applications of the method

CELLO-seq is a breakthrough method in the field of transposable element research, enabling identification of locus-specific expression of TEs at single-cell resolution. This will reveal information about the heterogeneity of transposable element expression across different cell types, in settings of both health and disease. CELLO-seq also enables exploration of locus-level TE heterogeneity within cells, allowing differentiation of transcripts derived from a highly repetitive fraction of the genome and an insight into locus-specific functions. Most significantly, CELLO-seq can uniquely identify transcripts from the youngest transposable elements, which remain under-represented in TE expression analyses to date. Not only is this insight locus-specific, but also allele- and isoform-specific. CELLO-seq enables detection of both TE and non-TE derived isoforms^19^. Cells derived from a variety of sources can be used for CELLO-seq, including cell lines and dissociated tissue. To date CELLO-seq has been used on fresh tissues such as early embryos and cryopreserved cell lines^19^. There is potential for application to frozen tissues, by performing conventional nuclei isolation on frozen tissues according to standard protocols for SMART-seq2^57,58^, and performing CELLO-seq on individual nuclei rather than cells. This expands the scope of CELLO-seq to understand expression of TEs at the individual locus level in preserved samples, for example, those belonging to patients. Additionally, the method holds promise for the virology field. Transcription of genetic elements derived from retroviruses can be detected in infected cells using CELLO-seq, with preliminary results suggesting the capability to look at the heterogeneity of transcripts derived from retroviral genetic elements across fixed or unfixed single cells (unpublished internal data).

### Limitations of the protocol

Unlike other single-cell methods, CELLO-seq requires a high number of PCR duplicates for error correction. Error correction uses a majority-based method to identify sequencing errors generated during ONT sequencing. PacBio sequencing could be used to avoid the error rate associated with ONT sequencing, however this has its own limitations including the inability to correct PCR errors. Furthermore, to achieve comparable depth with ONT sequencing, multiple cDNAs would need to be concatenated ahead of library preparation by combining CELLO-seq with MAS-ISO-seq.

Individual-specific variants in TE transcripts can only be identified if there is prior knowledge of where such integrants are within in a reference genome. This is not something that is provided in the CELLO-seq analysis pipeline by default, but it would be possible to identify integrants if they are overlapping genes or lead to read-through transcription. Expression of a full-length integration would only be identifiable in combination with long-read genomic DNA-seq of the same tissue or cell sample.

There are only a limited number of checkpoints within the protocol which can be used to determine whether reads of adequate length for TE sequencing, i.e. over 500bp. This means that RNA degradation will only be detected at late stages of the protocol leading to potential wasted time or reagents. Furthermore, even if RNA degradation has not occurred, not all reads will span the full length of the TE dependent on its length and whether reads are truncated.

The biggest limitation is the relatively low throughput of the method, a compromise necessary to enable the error correction and detection of young transposable elements. This is primarily a limitation of the sequencing depth of current long-read methods. However, it is possible to run several ONT PromethION flow cells, allowing for multiple CELLO-seq cDNA libraries to be sequenced simultaneously at sufficient depth. Furthermore, adjusting the volumes used in CELLO-seq to use 384-well plates as well as the possibility to use SPLiT-seq^59^ in combination with CELLO-seq, allowing the processing of several thousand cells at a time could be explored. Finally, CELLO-seq is optimised for stably transcribed TE RNAs and their isoforms which are polyadenylated. This means non-polyadenylated or nascent RNAs cannot be detected with CELLO-seq.

### Experimental Design

CELLO-seq is a plate-based sequencing method. A pre-requisite skill set of molecular biology, transcriptomics and data analysis is advised. We consider any scientist capable of performing plate-based sequencing methods such as SMART-seq to be able to perform CELLO-seq. Competency in R and use of genome browsers is recommended for downstream analyses beyond the bespoke sarlacc pipeline. A test CELLO-seq experiment can be performed to gain confidence in the technique by using 10 pg of purified RNA per well in an 8-well PCR strip, and scaling the reagents appropriately down from 96 wells, to 8 wells. This enables the user to test their own abilities and to ensure all the reagents are uncontaminated. As a readout, a bioanalyzer trace should show full-length cDNA.

**Figure 3:**
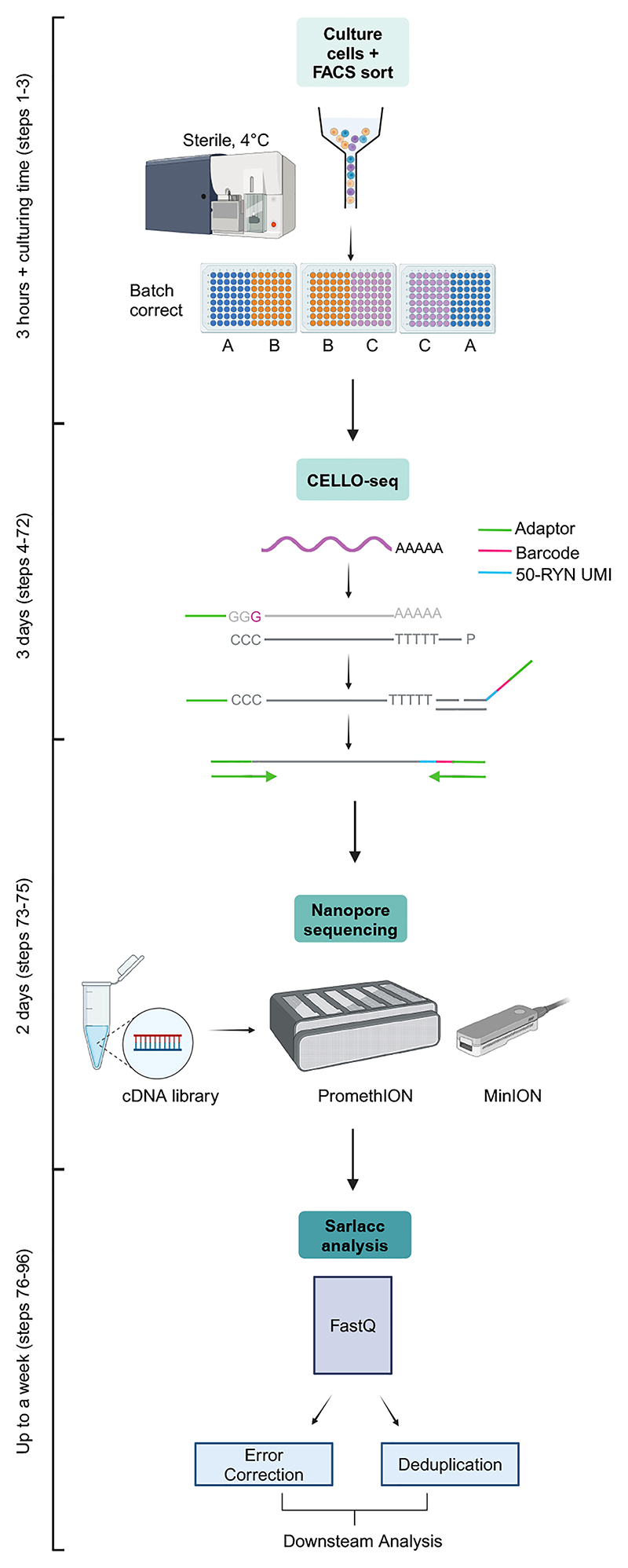
Flow chart for the experimental design of CELLO-seq along with suitable time frames for each stage. The protocol begins with the culturing and FACS sorting of cells of choice. CELLO-seq is then performed according to the protocol described, following by ONT sequencing. Finally, analysis of fastq files is performed via the sarlacc analysis pipeline on GitHub. Figure created with BioRender.com.

CELLO-seq requires individual cells to be FACS sorted into individual wells of a 96-well plate containing lysis buffer. It is possible to use 384-well plates if volumes are adjusted accordingly. Batch correction should be considered when carrying out CELLO-seq on multiple 96-well plates, and samples should therefore be distributed across replicate 96-well plates (Figure 3). Generally, at least two biological replicates per condition is recommended when looking to achieve statistical power. For homogenous cell types, 100 cells per condition is sufficient per biological replicate. However, for highly heterogeneous cell types containing small subpopulations, it is necessary to increase the cell number to have at least 50 cells for each respective cell type represented. Prior to commencing CELLO-seq, it may be desirable to ascertain whether TEs are likely to be expressed within cells of interest. Analysis of published RNA-seq datasets, qPCR for specific TE families, or immunofluorescent staining of TEs can act as an indicator of TE expression in different cells or tissues. After performing CELLO-seq, analysis of expression of cell-type specific genes is recommended to highlight the efficacy of the technique for detection of TE and gene expression.

#### Computational analysis with Sarlacc

**Figure 4:**
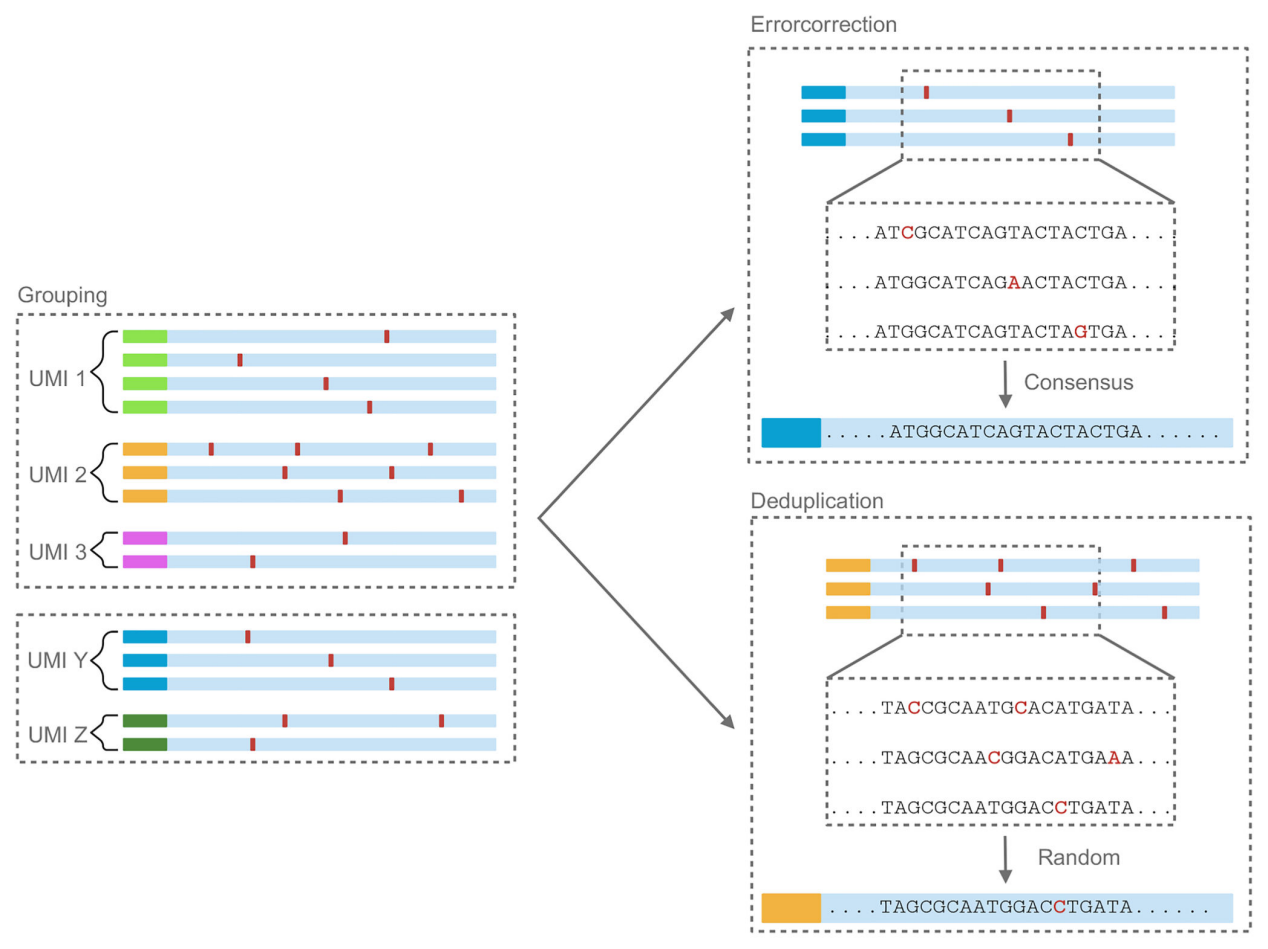
Schematic of CELLO-seq error correction and deduplication. After grouping reads to their transcriptome, reads are further grouped based on UMI. Reads can then be error corrected in each UMI group, whereby the consensus is taken by collapsing reads to omit sequencing errors. Alternatively, de-duplication mode can be used, where one of the reads is selected at random to represent the true sequence of the read. Figure created with BioRender.com.

Alongside the experimental protocol for CELLO-seq, a complimentary computational framework has been developed to error correct or de-duplicate each cDNA. This allows assignment of reads to their relevant TE locus of origin. The sarlacc computational workflow begins by demultiplexing ONT barcodes to assign reads to their respective cell. Pre-grouping of reads is then performed to align individual cDNAs to the transcriptome, improving efficiency and decreasing the computational power needed for subsequent error correction of reads per UMI sequence. A choice can be made by the user to either use error correction or de-duplication mode. For error correction, the consensus sequence is determined by multiple sequence alignment of up to 50 reads per UMI group. In de-duplication, a random read from the UMI group is selected to represent the sequence of the read (Figure 4). After creating consensus sequences, the final reads can be used for *de novo* transcriptome assembly. These transcriptomes can then be used to distinguish between genic and transposable element-derived transcripts. By overlaying the isoforms called in the *de novo* transcriptome assembly, TE-derived isoforms can be identified. The computational framework provides the user with counts tables of transcripts and reads, enabling visualisation and statistical analysis of transcription of unique TEs and genes. A small dataset from the provided to be used as a test for the scripts provided https://github.com/Berrenslab/CELLOseq_50ntUMI/blob/main/example%20data/E14CELLO_plate_8_50nt_test.fastq.gz

## Materials

### Biological Materials

Mammalian cells that have been freshly isolated, for example from dissociated tissue, or cultured after cryopreservation. Frozen tissue samples may also be used, however nuclear isolation from dissociated cells should be performed through applying standard nuclear isolation methods used for SMART-seq2^57,58^. Previous samples we have used for CELLO-seq include human induced pluripotent stem cells (iPS cells) (HPSI0714i-nufh_3, HPSI10914i-euts_1)^60^ and mouse blastomeres from female C57BL/6J mice.

### Caution

The use of human iPS cells and mouse embryos requires adherence to appropriate national laws and institutional regulatory board and funding agency guidelines. All iPS cell lines were obtained under appropriate material transfer agreements and approved by the institutional review board at Cambridge University. The mouse embryos were collected in accordance with the UK Home Office regulations and the Animals (Scientific Procedures) Act 1986.

### Reagents

- Ethanol absolute (Merck: 1.07017)
  **CAUTION:** Flammable, handle with care.
- Superase-In™ RNase Inhibitor (Thermo Fisher Scientific: AM2694)
- SuperScript™ IV Reverse Transcriptase kit (Invitrogen: 18090050)
  **CRITICAL**: We optimised the RT reaction substantially, including Betaine and ETSSB into the RT reaction, ensuring increased length and cDNA output.
- dNTP Set, 100mM Solutions (Thermo Fisher Scientific: R0182)
- HiFi Taq DNA Ligase (New England Biolabs: M0647S)
  **CRITICAL:** This ligase is required as it can be used at >50°C
- UltraPure™ DNase/RNase-Free Distilled Water (Invitrogen: 10977049)
- rCutSmart™ buffer (New England Biolabs: B6004S)
- Thermolabile Exonuclease I (New England Biolabs: M0568L)
- Kapa HiFi HotStart Uracil+ ReadyMix PCR Kit (Roche: KK2802)
- Betaine solution (5M; Sigma-Aldrich: B0300-5vl)
- ET SSB (New England Biolabs: M2401S)
  **CRITICAL:** we are not aware of a substitute item, but the ET SSB leads to an increase in cDNA length output
- Proteinase K (Molecular Biology Grade; New England Biolabs: P8107S)
  **CAUTION**: Proteinase K causes respiratory sensitisation. Handle it carefully using the appropriate safety equipment.
- Agencourt AMPure XP beads (Beckman Coulter: A63881)
- Evagreen Dye (Biotium: 31000-T)
- Triton X-100 (Sigma-Aldrich: T9284) **CAUTION:** Triton X-100 is harmful if swallowed. It causes serious eye damage. Handle it using appropriate safety equipment.
- DTT, Molecular Grade (Promega: P1171)
  **CAUTION:** DTT is toxic if swallowed. DTT causes skin corrosion and irritation as well as eye irritation and/or damage. Ensure the usage of appropriate safety equipment including eye protection and gloves.
- Invitrogen™ NaCl (5M), RNase-free (Invitrogen: AM9759)
- UltraPure™ 1M Tris-HCl, pH 8.0 (Invitrogen: 15568025)
- UltraPure™ 0.5M EDTA, pH 8.0 (Invitrogen: 15575020)
- TSO-RNA-Amine (IDT): 5’-TCGTCGGCAGCGTCAGATGTGTATAAGAGACArGrGrGAmn-3’
  **CRITICAL**: The Amine group on the 3’ end blocks the template switch oligo from acting as a primer.
- dT Oligo (IDT): 5’-PhosTGGCGTAGCGGGTTCGAGCGCACCGCAGGGTATCCGGCTATTTTTTTTTTTTTTTTTTTT TTTTTVN-3’
  **CRITICAL**: Phosphorylated dT oligo enables later splint oligo ligation.
- Top splint oligo (IDT): 5’-TAGCCGGATACCCTGCGGTGCGCTCGAACCCGCTACGCCATGTAAAACGACGGCCAGTInvd T-3’
  **CRITICAL**: The 3’ inverted dT (InvdT) in the top splint oligo leads to a 3’-3’ linkage with its adjacent nucleotide, inhibiting degradation by exonuclease I.
- Nextera R1 oligo (IDT): 5’-CGTCGGCAGCGTCAGATGTGTATAAGAGACAG-3’
- Nextera R2 oligo (IDT): 5’-GTCTCGTGGGCTCGGAGATGTGTATAAGAGACAG-3’
- Bottom splint (Splint oligos with UMI and ONT Barcodes (IDT): 5’-GTCTCGTGGGCTCGGAGATGTGTATAAGAGACAG-(ONTbarcode)-AGTGGTATCNRYNRYNRYNRYNRYNRYNRYNRYNRYNRYNRYNRYNRYNRYNRYNRYNRYA CTGGCCGTCGTTTTACATGGCGTAGCGGGTTCGAGCGCACCGCAGGGTATCCGGCTATTTTT TTTTTTTTTT-3’
  **CRITICAL**: The nucleotide length of the UMI (underlined) has been adjusted from 22 nt in our original publication^19^ to 50 nt to accommodate improved opportunities for error correction. 50 nt UMIs allow mapping of ambiguous TE loci with high sequence identity.

### Equipment

- Flow cytometry machine (BD Aria III, Becton Dickinson)
- pluriStrainer 40μm Cell Strainer (pluriSelect: 43-50040-51)
- Nunc™ Cell Culture Dishes (Thermo Fisher Scientific: 150318)
- DNA LoBind Tubes (1.5 ml, Eppendorf: 0030108051)
- Pipet-Lite Multi Pipette L8-10XLS+ (Rainin: 17013802)
- Pipet-Lite Multi Pipette L8-200XLS+ (Rainin: 17013805)
- Screw tube (50 ml, Sarstedt: 62.547.254)
- Mini vortexer (Varimix, SciQuip: SP2260-VM)
- Thermal cycler (C1000 Touch, Bio-Rad)
- DynaMag™-96 Side Skirted Magnet (Invitrogen: 12027)
- 0.2 ml 8-strip PCR tube Individually Attached Flat Caps (Starlab: A1402-3700)
- 96-well plate (Framestar: 4TI-0960)
- Adhesive PCR Plate Seals (Thermo Scientific: AB0558)
- Low retention LTS filter tips: 20, 200 and 1000 μl (Rainin: 30389226, 30389240, 30389213)
- DynaMag™-2 Magnet (Invitrogen: 12321D)
- Qubit assay tubes (Invitrogen: Q32856)
- Qubit dsDNA high-sensitivity (HS) kit (Invitrogen: Q32851)
- Qubit™ 4 Fluorometer, with WiFi (Invitrogen: Q33238)
- Agilent 2100 Bioanalyser (Agilent Technologies: G2938C)
- Agilent high-sensitivity DNA kit (Agilent Technologies: 5067-4626)
- 4200 TapeStation System (Agilent Technologies: G2991BA)
- High Sensitivity D5000 ScreenTape (Agilent Technologies: 5067-5592)
- High Sensitivity D5000 Reagents (Agilent Technologies: 5067-5593)
- Benchtop centrifuge (510R, Eppendorf)
- Mini microcentrifuge (Corning: 6770)
- CoolRack XT PCR96 Thermoconductive Tube Rack for 96-Well PCR Plates (Azenta Life Sciences: BCS-529)
- CoolBox XT All-day Cooling and Freezing Workstation, Single Capacity (Corning: 432021)
- RNase*Zap*™ RNase Decontamination Solution Catalog number (Invitrogen: AM9780)

### Hardware and Software requirements

**Figure 5:**
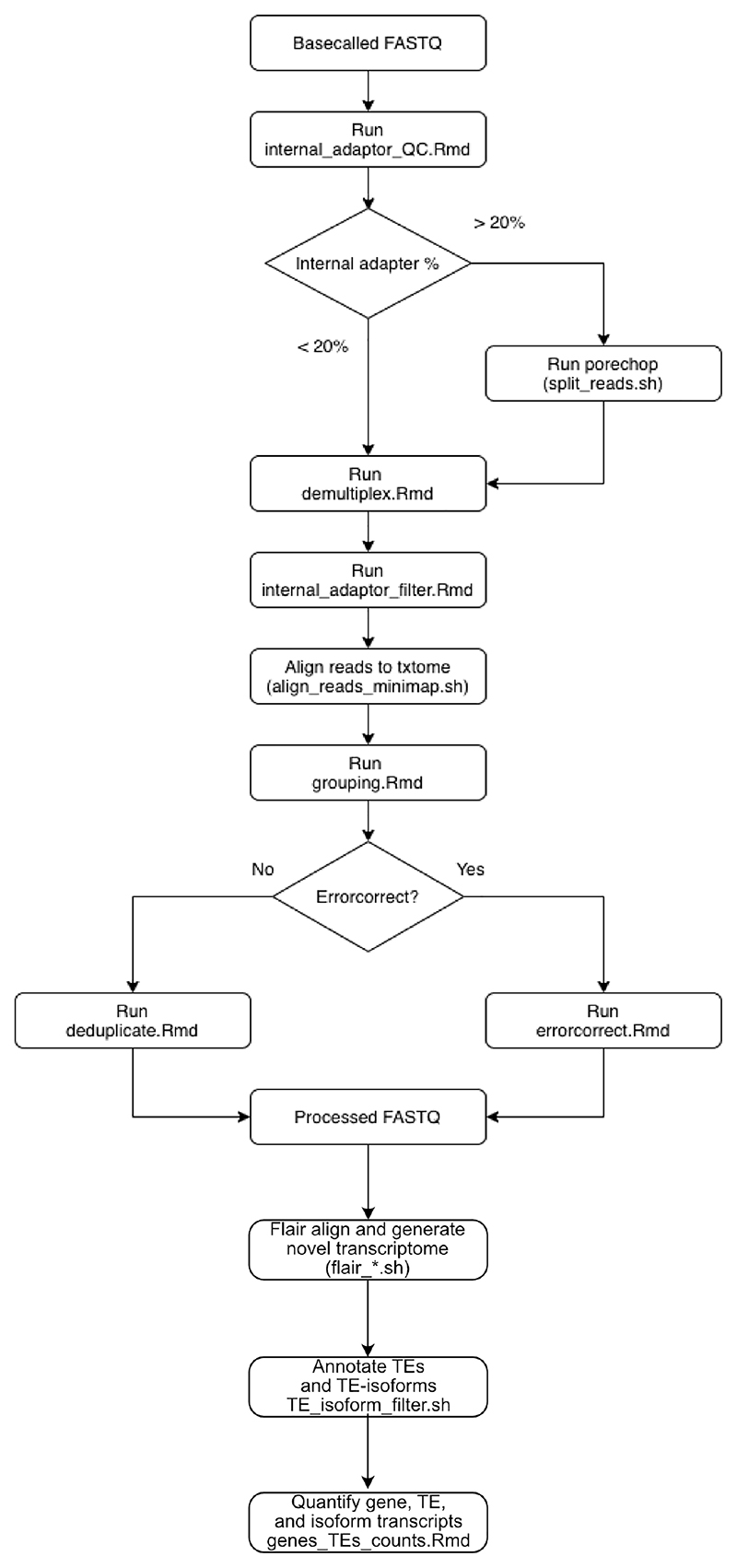
Flowchart of CELLO-seq analysis pipeline. Basecalled fastq are screened for internal adaptor sequences. Based on the percentage of reads containing internal adaptors, these can be computationally split to extract separate reads with porechop (see Troubleshooting). Quality checked fastq are demultiplexed, generating fastq for individual ONT barcodes. Internal adaptor containing reads are filtered prior to alignment to the transcriptome with minimap2. Aligned reads are grouped by transcript sequence prior to error correction. Transcripts are error corrected, producing consensus sequences for each UMI. Error corrected fastq are then used for generation of a de novo transcriptome assembly with FLAIR. FLAIR alignment and quantification generates a table containing each transcript and the number of reads per cell. TEs and isoforms are annotated with genomic location using TE_isoform_filter.sh and genes_TEs_counts.Rmd, with the option of generating SCE objects for compatibility with downstream single-cell RNA-seq analysis tools in R. Figure adapted with permission from ref.^19^, Springer Nature.

- R based computing is necessary for CELLO-seq analysis and can be carried out locally or through high performance computing clusters.
- Basecalling of raw ONT reads can be performed using the super high accuracy model implemented in Guppy (v.6.4.2) or the Super accurate (SUP) model in Dorado (v.4.3.0).
- CELLO-seq data processing is performed using a computational pipeline in R (Figure 5), Sarlacc (https://github.com/MarioniLab/sarlacc). CELLO-seq specific modifications to Sarlacc allow ONT barcodes demultiplexing, adaptor trimming, and UMI identification for error correction or deduplication of each cDNA molecule. Code can be found at (https://github.com/MarioniLab/CELLOseq/tree/master/pipeline). Sarlacc is available as a singularity image at (https://github.com/Berrenslab/CELLOseq_50ntUMI/blob/main/images/sarlacc.img.gz).
- Reads containing internal adaptor sequences can be split using Porechop. Porechop is available as a singularity image at: (https://github.com/Berrenslab/CELLOseq_50ntUMI/blob/main/images/porechop.img.gz).
- Data pre-processing steps also require minimap2(v2.17)^61^, samtools (v1.17)^62^, and bedtools^63^. Installation information can be found at: https://github.com/lh3/minimap2/releases/tag/v2.17, https://bedtools.readthedocs.io/en/latest/content/installation.html#, and https://github.com/samtools/samtools/releases/tag/1.17.
- Generation of the de-novo transcriptome and final read alignments is performed using flair. A version-controlled CELLO-seq compatible version of flair can be found at (https://github.com/Berrenslab/CELLOseq_50ntUMI/blob/main/images/flairdev.img.gz).
- Data visualisation software such as IGV^64^ or JBrowse2^65^ is required for visualisation of bam file outputs from flair. Quantitative analysis in R is recommended using Bioconductor packages such as SingleCellExperiment and scRNAseq from Seurat^66^.

### Reagent setup

#### Cell lysis buffer

Combine 0.2% (vol/vol) Triton X-100 and 1 U/μl RNase inhibitor (such as SUPERase-In) and aliquot into a 96-well plate at 2 μl per well. These 96-well plates can be covered with adhesive PCR film and stored at -80°C for up to 2 years.

**CRITICAL**: Lysis buffer preparation should be carried out in a UV-treated hood. The lysis buffer should be pipetted into a 96-well plate on a cold block to ensure activity of the RNase inhibitor is retained.

#### TSO-RNA-Amine (IDT)

Use High Performance Liquid Chromatography (HPLC) purification to retrieve full length oligos. Store in 12 ul aliquots at -80C for up to 2 years.

#### dT oligo (IDT)

Use HPLC purification to retrieve full length oligos. Store in 110 μl aliquots at -20°C for up to 2 years.

#### Top splint oligo (IDT)

Use HPLC purification to retrieve full length oligos. This can be stored at -20°C for up to 2 years.

#### Nextera R1 oligo (IDT)

Perform standard desalting before use. This can be stored at -20°C for up to 2 years.

#### Nextera R2 oligo (IDT)

Perform standard desalting before use. This can be stored at -20°C for up to 2 years.

#### Bottom splint (Splint oligos with UMI and ONT Barcodes (IDT)

Use HPLC purification to retrieve full length oligos. This can be stored at -20°C for up to 2 years.

#### Oligo hybridisation buffer

Prepare 10x buffer by combining 500 mM NaCl, 10 mM Tris-Cl pH 8.0, 1 mM EDTA pH 8.0 in nuclease-free water. This can be stored at -20°C for up to 2 years.

### Procedure

#### Single-cell sample preparation. Timing: Up to 3 h

**CRITICAL:** Lysis buffer preparation and all subsequent steps of CELLO-seq should be carried out in a UV-treated hood, with any equipment entering the hood to be thoroughly wiped down with RNase zap. The lysis buffer should be pipetted into a 96-well plate on a cold block to ensure activity of the RNase inhibitor is retained.

1) Prepare the required number of 96-well plate containing cell lysis buffer as specified in the Reagent Setup section. The volumes should be scaled up appropriately for the number of reactions required, and then individual reagent volumes further scaled up by 1.1x to account for pipetting errors.

2) Different sources of starting cells can be used to perform CELLO-seq, including cultured cells or cells dissociated from tissues. Wash with PBS and filter with a cell strainer to remove cell clumps prior to FACS sorting. Frozen tissue samples may also be used, however nuclear isolation from dissociated cells should be performed through applying standard nuclear isolation methods used for SMART-seq2^57,58^.

3) Stain live cells with 1 μg/μl DAPI and sort into individual wells of a 96-well plate containing lysis buffer through carrying out FACS, with the FACS machine positioned in a sterile hood.

**CRITICAL STEP:** The FACS machine must be in a sterile hood with laminar flow to prevent contamination during cell sorting. Additionally, always keep the 96-well plate with lysis buffer on a cold block, at 4°C whenever it is not inside the FACS machine.

**PAUSE POINT:** 96-well plates containing cell lysates can be stored at -80°C for later processing for up to 2 years.

#### RNA annealing. Timing: 20 min

**CRITICAL:** Throughout CELLO-seq, the 96-well plate is placed on a cool block when otherwise not in the thermocycler or plate centrifuge.

**CRITICAL:** Throughout the CELLO-seq protocol all reagents must be thawed on ice.

4) Prepare an annealing mix according to the table below.

**Table:** 

Component	Volume (μl)	Final concentration in 96-well plate
dT oligo (100 μM)	1	23.80 μM
dNTPs (10 mM)	1	2.38 mM
SuperaseIN (20 U/μl)	0.2	0.95 U/μl

5) Add 2.2 μl of the RNA annealing mix to each well of the 96-well plate, which already contains 2 μl of cell lysate per well.

6) Vortex the 96-well plate and spin down for 1 min at 1000g in a plate centrifuge cooled to 4°C.

7) Pre-heat the thermocycler to 72°C.

**CRITICAL STEP:**All thermocycler settings are carried out with a lid temperature 5°C greater than the maximum temperature during a thermocycler incubation.

8) Incubate plate according to the conditions in the table below:

**Table:** 

Cycle	Temperature (°C)	Time
1	72	3 min
2	4	Hold

9) Spin down the plate for 1 min at 1000g in a plate centrifuge cooled to 4°C.

#### Reverse Transcription. Timing: 2.5 h

10) Prepare the RT mix as following. Note that SuperScript IV buffer (SSIV buffer) can be found in the SuperScript IV Reverse Transcriptase kit:

**Table:** 

Component	Volume (μl)	Final concentration in 96-well plate
Nuclease-free water	1.6	-
SSIV buffer (5x)	2	1x
Betaine (5 M)	1	0.5 M
TSO (100 μM)	0.1	1 μM
DTT (100 mM)	0.5	5 mM
Superscript IV (200 U/μl)	0.5	10 U/μl
ET SSB (500 μg/ml)	0.1	5 μg/ml

11) Pipette 5.8 μl of the RT mix into each well of the 96-well plate.

12) Vortex the 96-well plate and spin down for 1 min at 1000g in a plate centrifuge cooled to 4°C.

13) Preheat the thermocycler to 53°C.

14) Place the plate into the thermocycler and incubate according to the following program:

**Table:** 

Cycle	Temperature (°C)	Time
1	53	10 min
2	42	2 h
3	4	Hold

15) Return plate to cold block.

16) Spin down the plate for 1 min at 1000g in a plate centrifuge cooled to 4°C.

#### Exonuclease I treatment. Timing: 1.5 h

17) Prepare the Exonuclease I mix by combining and mixing by pipetting the following components:

**Table:** 

Component	Volume (μl)	Final concentration in 96-well plate
Nuclease-free water	4	-
CutSmart (10x)	0.5	0.33x
Exonuclease I (20 U/μl)	0.5	0.67 U/μl

18) Pipette 5 μl of Exonuclease I mix per well of the 96-well plate.

19) Vortex plate and spin down for 1 min at 1000g in a plate centrifuge cooled to 4°C.

20) Return plate to the cold block and pre-heat the thermocycler to 37°C.

21) Incubate in the thermocycler according to the following program:

**Table:** 

Cycle	Temperature (°C)	Time
1	37	1 h
2	95	3 min
3	4	Hold

22) Spin down the plate for 1 min at 1000g in a plate centrifuge cooled to 4°C.

**PAUSE POINT**: If necessary, the reaction can be paused here and kept at 4°C for up to 7 days.

#### Preparation of splint oligos. Timing: 45 min

23) Prepare the splint hybridisation mix by combining the following components:

**Table:** 

Component	Volume (μl)	Final concentration in 96-well plate
Nuclease-free water	7	-
Oligo hybridisation buffer (10x)	1	0.4x
Top splint (100 μM)	1	4 μM

24) To each well of a new 96-well plate placed on a cold block, pipette 9 μl of the splint hybridisation mix.

25) Add 1 μl of each bottom splint oligo to individual wells of the 96-well plate.

**CRITICAL STEP:** Each bottom splint should be unique in each well of the 96-well plate.

26) Hybridise the top and bottom split oligos through following the thermocycler program below:

**Table:** 

Cycle	Temperature (°C)	Time	Comments
1	95	1 min	
2-82	95-15	20 s	Decrease by 1°C per cycle
83	4	Hold	

27) Spin down the plate for 1 min at 1000g in a plate centrifuge cooled to 4°C and return to cold block.

**CRITICAL STEP**: Preparation of splint oligos in this manner will yield enough to perform CELLO-seq on ten 96-well plates.

**CRITICAL STEP**: Once splint oligos are prepared, they should be kept on ice if being used imminently.

**PAUSE POINT**: If not being used immediately, splint oligos can be stored at -20°C for up to 2 years.

#### Ligate the splint oligo onto cDNA. Timing: 1.5 h

28) Prepare the splint oligo ligation mix as follows by combining the following reagents and mixing by careful pipetting:

**Table:** 

Component	Volume (pl)	Final concentration in 96-well plate
Nuclease-free water	3	-
HiFi Taq ligase buffer (10x)	0.5	0.17x
HiFi Taq ligase	0.5	-

**CRITICAL STEP:** Thaw HiFi Taq ligase buffer at room temperature (20-25°C) and mix thoroughly by vortexing prior to preparing the splint oligo ligation mix.

29) Pipette 1 μl of each unique annealed splint oligo to a different well of the original 96-well plate that has undergone exonuclease I treatment.

30) Pipette 4 μl of the splint oligo ligation mix to each well of the 96-well plate.

**CRITICAL STEP:** Ensure that splint oligos are pipetted into each well of the 96-well plate prior to the mastermix to prevent premature ligation of oligos to the cDNAs.

31) Vortex plate and spin down for 1 min at 1000g in a plate centrifuge cooled to 4°C.

32) Pre-heat the thermocycler to 55°C and perform the splint oligo ligation reaction by performing the following thermo cycle incubation:

**Table:** 

Cycle	Temperature (°C)	Time
1	55	1 h
2	4	Hold

33) Spin down the plate for 1 min at 1000g in a plate centrifuge cooled to 4°C.

#### Proteinase K digestion. Timing: 20 min

**CRITICAL**: Proteins leftover from earlier reactions must be digested with proteinase K as they are sticky and will build up on beads used in the AMPure XP cleanup (steps 39 to 51).

34) Prepare a proteinase K mix should by combining the following reagents:

**Table:** 

Component	Volume (μl)	Final concentration in 96-well plate
Nuclease-free water	4.83	-
Proteinase K (800 U/ml)	0.17	4 U/ml

35) Pipette 5 μl into each well of the 96-well plate.

36) Vortex and spin down the plate for 1 min at 1000g in a plate centrifuge cooled to 4°C.

37) Pre-heat thermocycler with a heated lid to 50°C and incubate the reaction according to the following program:

**Table:** 

Cycle	Temperature (°C)	Time
1	50	5 min
2	4	Hold

38) Spin down the plate for 1 min at 1000g in a plate centrifuge cooled to 4°C and return to cold block.

#### AMPure XP cleanup. Timing: 1 h

39) Before starting the cDNA clean-up, equilibrate AMPure XP beads at room temperature for 30 mins and vortex thoroughly until the liquid is homogeneous.

40) Prepare 30 ml of 80% ethanol by diluting 24 ml of pure ethanol in 6 ml of nuclease-free water in a tube.

41) Add 12.5 μl of beads directly into each 25 μl sample (1:2 ratio) in the 96-well plate and mix well by pipetting up and down 10 times, being careful to not introduce bubbles.

42) Incubate the 96-well plate for 5 min at room temperature.

43) Place the plate on the magnetic plate rack and incubate for 5 min at room temperature.

44) Pipette off liquid from each of the wells of the 96-well plate.

45) Pour the 80% ethanol into a clean, nuclease-free reservoir and whilst the 96-well plate is on the magnetic plate rack, pipette 150 μl per well of the 96-well plate using an 8-channel pipette and pipette-off and discard the liquid.

46) Pipette 150 μl of ethanol again per well and pipette off.

47) Centrifuge for 1 min at 1000g in a plate centrifuge cooled to 4 °C.

48) Dry the beads for 3 min approx., when the beads turn from shiny to matte.

**CRITICAL STEP**: Ensure that the beads are not cracked in appearance, this indicates over-drying and compromises the yield of cDNA retrieved.

49) Take the 96-well plate off the magnetic plate rack and resuspend in 6.5 μl of nuclease-free water per well of the 96-well plate and let incubate for 5 min.

50) Place the 96-well plate back on the magnetic plate rack and incubate for 5 min.

51) Carefully pipette the contents of each well of the 96-well plate into a fresh 96-well plate, ensuring that any beads are left behind.

**PAUSE POINT:** The cDNA in the 96-well plate can now be covered with PCR-film and stored for up to 7 days at 4°C or up to 4 weeks at -20°C.

#### Test amplification qPCR. Timing: 5.5 h

52) Prepare the qPCR mix as follows:

**Table:** 

Component	Volume (μl)	Final concentration in 96-well plate
Nextera R1 primer (100 μM)	0.625	2.5 μM
Nextera R2 primer (100 μM)	0.625	2.5 μM
Kapa HiFi HotStart Uracil (2x)	12.5	1x
Betaine (5 M)	5	1 M
ET SSB	0.25	-
Nuclease-free water	4.38	-
Evagreen (20x)	1.25	1x

53) Pipette 0.37 μl of cDNA from each well of the 96-well plate from the AMPure XP clean-up into a fresh 96-well PCR plate and pipette 24.5 μl of the qPCR mix into each well.

54) Seal the PCR plate with an adhesive PCR plate seal and vortex the plate for a few seconds to mix and spin down for 1 min at 1000g in a plate centrifuge cooled down to 4°C.

55) Place the plate in a thermocycler and carry out qPCR according to the conditions in the table below:

**Table:** 

Cycle	Denature	Anneal	Extend	Final Extension	Hold
1	90°C, 3 min	-	-		-
2-40	90°C, 20 s	65°C, 30 s	72°C, 10 min		-
41				72°C, 3 min	
42					4°C

56) Choose the correct number of PCR cycles for CELLO-seq without overamplification. This is equivalent to choosing 3 cycles before exponential growth in the logarithmic phase on the qPCR trace. It would be expected that 25-29 cycles would be appropriate for PCR.

**CRITICAL STEP:** Choosing the appropriate number of PCR cycles is crucial to allow for error correction of transcriptomic libraries. Too few cycles will reduce the number of PCR duplicates generated, which limits the ability to error correct, whilst too many cycles will result in overamplification.

#### PCR. Timing: 5.5 h

57) Prepare a PCR mix according to the following table:

**Table:** 

Component	Volume (μl)	Final concentration in 96-well plate
Nextera R1 primer (100 μM)	0.625	2.5 μM
Nextera R2 primer (100 μM)	0.625	2.5 μM
Kapa HiFi HotStart Uracil (2x)	12.5	1x
Betaine (5 M)	5	1 M
ET SSB	0.25	-

58) Pipette 19 μl of the PCR mix into each well of the 96-well plate, containing the remaining 6 μl of cDNA.

59) Vortex plate for a few seconds and spin down for 1 min at 1000g in a plate centrifuge cooled down to 4°C.

60) Place the plate in a thermocycler and carry out PCR according to the conditions in the table below:

**Table:** 

Cycle	Denature	Anneal	Extend	Final Extension	Hold
1	90°C, 3 min	-	-		-
2-x, depending on qPCR	90°C, 20 s	65°C, 30 s	72°C, 10 min		-
x+1				72°C, 3 min	
x+2					4°C

61) When the PCR reaction has finished, spin down the 96-well plate in a plate centrifuge cooled down to 4°C at 1000g.

#### AMPure XP Cleanup. Timing: 1 h

**CRITICAL** After PCR, all subsequent steps of the protocol can be performed outside of a UV-treated flow hood.

62) Equilibrate AMPure XP beads to room temperature and vortex the beads thoroughly so that they are a liquid homogenous in colour.

63) Pool together the PCR reactions from all wells of the 96-well plate into three 1.5 ml LoBind Eppendorf tubes. Ensure that there are equal volumes of the pooled sample in each of the tubes.

64) Prepare 6 ml of 80% ethanol by diluting pure ethanol in nuclease-free water.

65) Pipette one volume of AMPure XP beads to every 2 volumes of pooled PCR reaction. Mix by pipetting up and down 10 times, being careful not to introduce bubbles and let the mixture incubate at room temperature for 5 min. For each of the three Eppendorf tubes, perform the following:

- a) Place the Eppendorf tube on a magnetic rack and allow to incubate for 5 min at room temperature.
- b) Pipette off and discard the liquid from the Eppendorf tube and add the volume of 80% ethanol which allows the AMPure XP beads to be submerged.
- c) Pipette off and discard the ethanol from the Eppendorf tube and spin down the tube in a mini microcentrifuge.
- d) Place the Eppendorf tube back on the magnetic rack and pipette off and discard any excess ethanol and allow the AMPure XP beads to dry for approximately 10 mins, or until they turn from shiny to a matte brown colour.
- e) Take the Eppendorf tube off the magnetic rack and resuspend the AMPure XP beads in 30 μl of nuclease-free water per tube and incubate at room temperature for 5 min.

66) Place each of the three Eppendorf tubes back onto the magnetic rack and incubate for 5 min at room temperature.

67) Carefully pipette the liquid from each of the three Eppendorf tubes, merging the liquid into a new LoBind Eppendorf tube, ensuring beads are not present.

68) Measure the volume of liquid in the single LoBind Eppendorf tube, this should be slightly below 90 μl. Pipette 1 volume of AMPure XP beads to 2 volumes of measured liquid. Mix by pipetting up and down 10 times.

69) Repeat steps 65 a) to d).

70) Take the Eppendorf tubes off the magnetic rack and resuspend the AMPure XP beads in 55 μl of nuclease-free water per tube and incubate at room temperature for 5 min.

71) Place the Eppendorf tube back on the magnetic rack and incubate for 5 min at room temperature.

72) Carefully pipette the liquid, containing the cDNA library, into a fresh LoBind Eppendorf tube, being careful to avoid any AMPure XP beads.

**PAUSE POINT:** The cDNA in the LoBind Eppendorf tube can and stored for up to 7 days at 4°C or up to 4 weeks at -20°C.

#### Quantifying DNA length and concentration. Timing: 1 h

**Figure 6:**
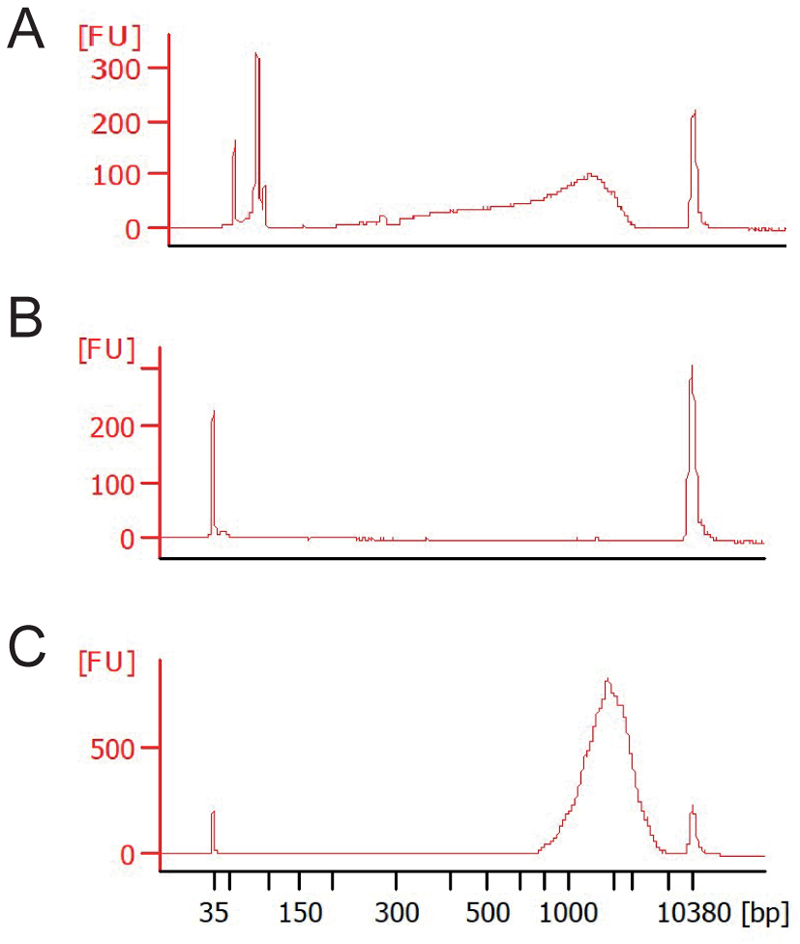
Example fragment analysis after CELLO-seq. Post-amplification Bioanalyzer traces of cDNA libraries from mESCs after two AMPure XP clean-ups. (A) Example Bioanalyzer trace with a broad peak spanning ~200-1000 bp, characteristic of RNA degradation during cDNA library preparation. The sharp peak at ~100 bp is due to the formation of primer dimers. (B) Bioanalyzer trace after unsuccessful cDNA amplification, indicative of no cell present. (C) An example Bioanalyzer trace after a successful round of CELLO-seq, with one clear peak at ~2000 bp and no evidence of RNA degradation.

73) Using a Bioanalyser or TapeStation, quantify the average length of DNA in the transcriptomic library (Figure 6), as well as the concentration using the Qubit, according to manufacturer’s instructions.

#### ?Troubleshooting

74) Using molarity, calculate the appropriate dilution required to give 200 fmol of transcriptomic library.

#### ONT library preparation. Timing: 2 d

75) Perform ONT library preparation according to manufacturer’s instructions for the appropriate Ligation Sequencing Kit of the ONT sequencing run selected; a ONT sequencing run using a PromethION sequencer and a R10 flow cell run leads to 40-80 million reads obtained per flow cell. Alternatively, ONT sequencing can be carried out by using a MinION flow cell on a MinION sequencer and will yield approximately 10,000 reads per single cell.

**CRITICAL STEP:** The decision to use a MinION or PromethION flow cell and sequencer is entirely based on the sequencing depth the user hopes to achieve. We recommend using a PromethION flow cell and sequencer if sequencing more than 10 cells to obtain optimal sequencing depth. Otherwise, sequencing with a MinION is sufficient.

## Data analysis. Timing: 1 week

**CRITICAL** Steps 77-88 describe CELLO-seq data analysis using Sarlacc (Figure 5), while steps 89-95 describe analysis using FLAIR.

**CRITICAL** A small test dataset of 100,000 basecalled reads is available at (https://github.com/Berrenslab/CELLOseq_50ntUMI/blob/main/example%20data/E14CELLO_plate_8_50nt_test.fastq.gz) for trialling the computational framework. Downstream processing can then be undertaken with examples in as relevant to user needs.

76) Remove reads greater than 20kb with filter_read_lengths.sh due to their incompatibility with the Biostringr package.

77) The first step in the pipeline is to perform quality control analysis of the reads and of the adaptors. To do this, run the internal_adaptor_QC.Rmd script.

78) Run the internal_adaptor_QC.Rmd script again, as this script is designed to analyse each adaptor separately, so needs to be run twice (ideally in parallel to reduce runtime). If run successfully this will output:

∘ The number of reads
∘ A histogram of read lengths
∘ Histograms of alignment scores for adaptors against the read sequences
∘ The percentage of reads containing internal adaptors due to concatenation during library preparation (defined as within 20%-80% of the read length).

### ?Troubleshooting

**Figure 7:**
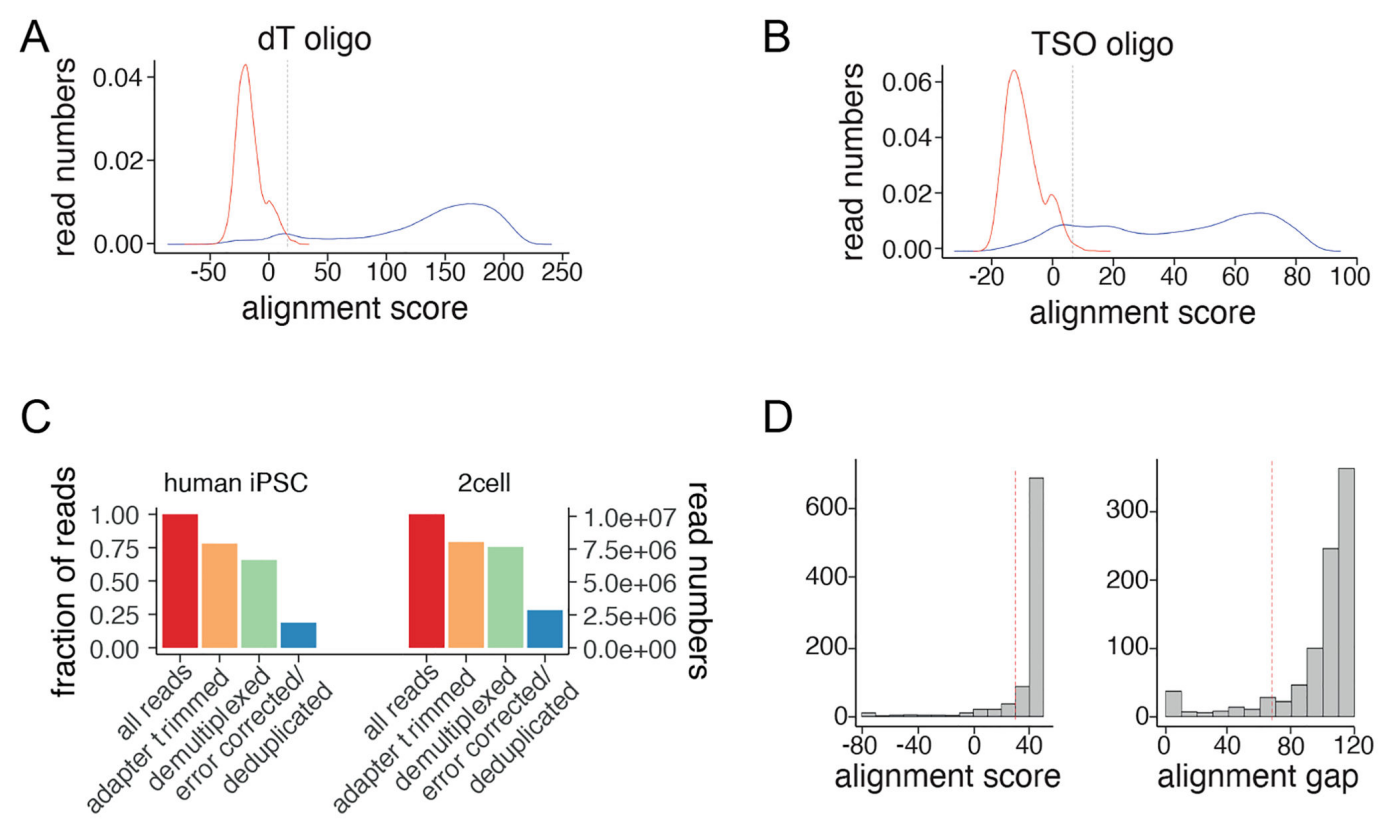
Computational quality control of CELLO-seq libraries with Sarlacc. Alignment scores of real (blue) and scrambled (red) reads to dT oligo (A) and tso oligo (B) in internal adaptor QC. (C) Bar plot showing the number of raw reads generated for one MinION flow cell (all reads), number of reads with adapters on both ends (adapter trimmed), number of reads assigned to ONT barcodes (demultiplexed) and molecules per MinION flow cell (error corrected/deduplicated) for 22nt UMI human iPSC (left) and 22nt UMI mouse 2cell data (right). (D) Histogram of alignment scores and alignment gap scores for alignment of reads to ONT barcode sequences from demultiplexing. Dotted lines show thresholds used for filtering of false barcode sequences. Panels A and B generated using test dataset of 1 million reads from 2-cell 22nt UMI CELLO-seq dataset available at (https://github.com/Berrenslab/CELLOseq_50ntUMI/tree/main/example%20data/E14CELLO_plate_8_50nt_test.fastq.gz). Panel D for data generated from a 50nt UMI library, sequenced on a R10 PromethION flow cell with ligation sequencing kit 114.

79) After the initial quality control, we perform demultiplexing of reads by alignment to the sample ONT barcodes with demultiplex.Rmd. The alignment scores help to ensure correct adaptor usage and ligation (Figure 7A and B).

80) Define the barcode sequences that could be present in the first stretch of ’N’s in ’adaptor1’. We have included the 96 barcode sequences from ONT in the script, though it is recommended that you include only the barcode used in the experiment.

81) Define the filepath to the fastq in the script to enable adaptor alignment:

aln.out <-adaptorAlign(adaptor1, adaptor2, filepath=“path/to/.fastq”, gapOpening=4, gapExtension=1)

**CRITICAL STEP:** By default, the alignment will only consider the 250 bp on either end of the read to reduce the runtime, under the assumption that the adaptors should occur at the read ends. This can be further sped up by parallelising jobs using the ’BiocParallel^67^‘ package with the appropriate backend supplied to the optional ’BPPARAM=‘ argument.

82) Run the demultiplex.Rmd script, which will retain only reads that were unambiguously assigned to a barcode. It will output:

∘ demultiplexed reads by sample as a fastq file for the alignment step
∘ a rds file containing the adaptor alignment information (for use in grouping and error correction/deduplication).

83) Filter demultiplexed reads for those containing internal adaptors using internal_adaptor_filter.Rmd.

84) As with the internal_adaptor_QC.Rmd, run the internal_adaptor_filter.Rmd again, as this script is also designed to analyse each adaptor separately, so needs to be run twice. The adaptor must be defined along with the path to fastq as in step 78.

85) Use the dispatcher_internal_adaptor_filter.R to read in the set of fastq generated during demultiplexing.

86) Align filtered reads to the reference genome using minimap2^61^ in the align_reads_minimap.sh script. This will output a .sam file for each barcode.

87) Following alignment of the reads against the transcriptome, use the grouping.Rmd script to pre-group reads based on transcript/repeat by clustering similar UMI sequence together. This generates read groups for error correction or de-duplication. Since error correction can take some time to run, the script splits the list of read groups into multiple smaller chunks and limit the amount of reads per groups. Run time can be reduced by parallelisation of this step using the dispatcher_grouping.R script.

88) After UMI grouping, either perform error correction or deduplication to collapse each read groups into a single read. Scripts can be run on all files using the dispatcher_*.R files.

- a) In the deduplicate step, we randomly select a read from each group to select as the representative read.
- b) In the error correction step, we perform multiple sequence alignment within read groups and use the consensus sequence as the representative read.

89) Once fastq have been obtained, perform downstream analysis of CELLO-seq using FLAIR^68^ (https://github.com/Berrenslab/CELLOseq_50ntUMI/blob/main/images/flairdev.img.gz) as described in the following steps, or using similar methods for *de novo* or transcriptome guided transcriptome assembly.

90) First, align reads to the reference genome using minimap2 in flair_align.sh. Further instructions for reference genomes and custom annotations at https://github.com/Berrenslab/CELLOseq_50ntUMI/tree/main/README_referencegenomes.txt.

### ?Troubleshooting

**Figure 8:**
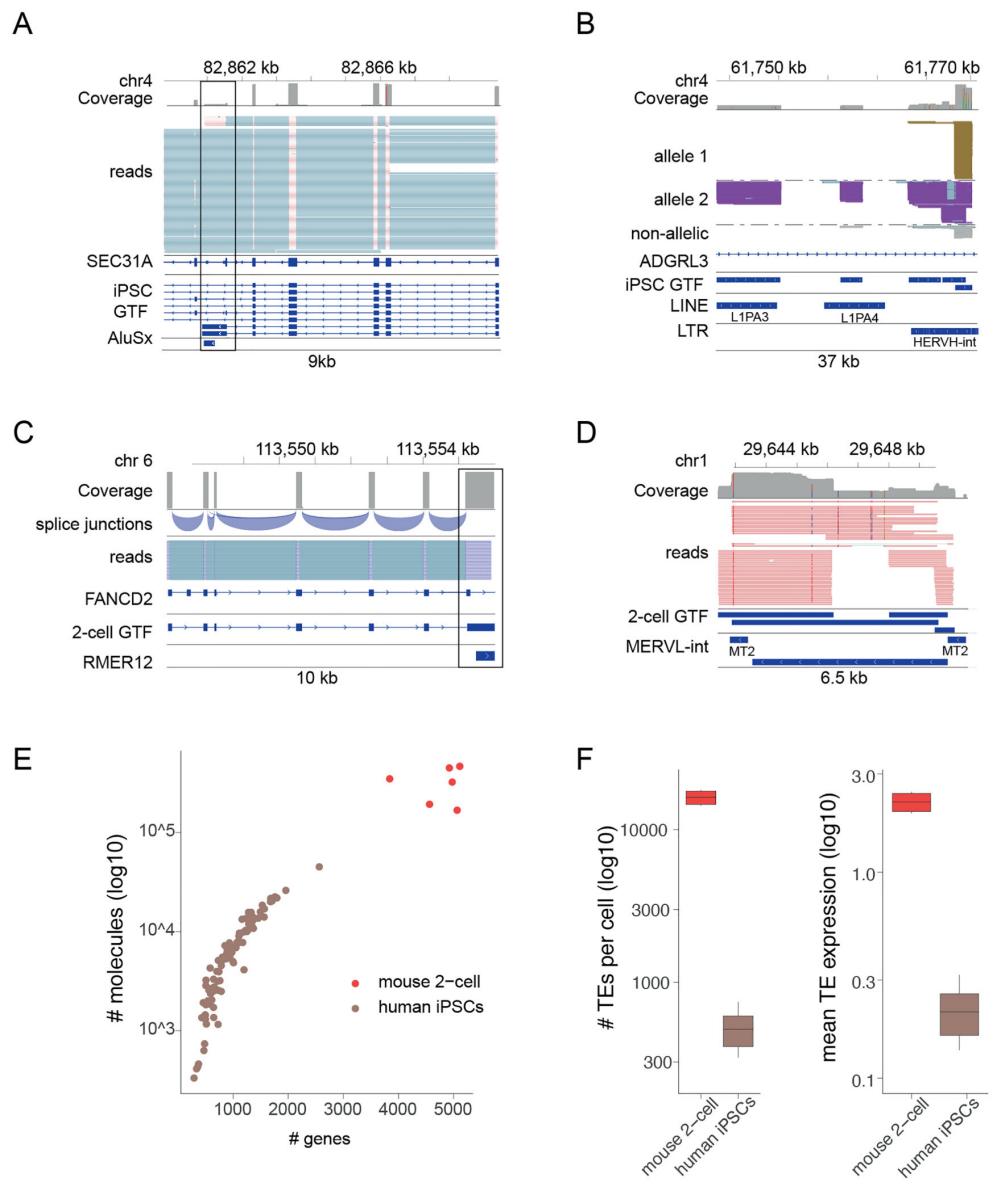
Allocation of reads to TEs and genes and the numbers expected per cell type. Example genome browser depicting error corrected reads from human iPSCs aligned to a human gene and TE isoform (A), and a L1PA3,4 and HERVH-int (B). Example genome browser view depicting error corrected mouse 2-cell stage embryo reads aligned to a mouse gene and a TE isoform (C) and a MERVL (D). (E) A scatterplot of molecules (y-axis) per genes (x-axis) in mouse two-cell blastomeres (red) and hiPSCs, (brown). (F) Boxplots for mouse two-cell blastomeres (red) and hiPSCs (brown) on the x-axis, against the number of autonomous TEs per cell (left) and the mean expression of autonomous TEs (right). Boxplots show the median, first and third quartiles as a box, and the whiskers indicate the most extreme data point within 1.5 lengths of the box. Y-axes on a log10 scale. Mouse two-cell blastomeres, n=6 cells and hiPSCs n= 96 cells. Both datasets generated using 22nt UMIs. For panel F boxplots, the median, first and third quartiles are a box and the whiskers indicate the most extreme data point within 1.5 lengths of the box. Figure reproduced with permission from ref.^19^, Springer Nature.

91) Next, correct misaligned splice sites using both the genome exon annotations and short-read splice junctions in flair_correct.sh.

92) Run flair_collapse.sh, to define high-confidence isoforms from all the corrected reads.

93) Finally, quantify FLAIR isoform usage and gene and TE counts across samples by running flair_quantify.sh. This produces a *de novo* transcriptome assembly in the form of a GTF file. Alongside the bam output (Figure 8A-D) from flair_align.sh, this can be visualised in a genome browser such as IGV.

94) From the FLAIR quantify output, annotate genes, TEs and TE isoforms using bedtool intersect with known repeats, known isoforms and overlap between novel isoforms and TEs, respectively using the bash script TE_isoform_filter.sh.

95) Based on user requirements, merge these annotations with the FLAIR quantify output in genes_TE_counts.Rmd. This generates counts tables containing quantified transcript and location information for TEs, TE-isoforms, and genes.

96) **(Optional)** Follow SingleCellExperiment Bioconductor package for single-cell experiment object generation compatible with further single-cell RNA-seq analysis tools. This will generate data arrays which can be visualised in R (Figure 8E-F).

### Troubleshooting

Troubleshooting advice can be found in Table 1

### Timing Day 1

Steps 1-3, Single-cell sample preparation: ~3 h

### Day 2

Steps 4-9, RNA annealing: 20 min

Steps 10-16, Reverse Transcription: 2.5 h

Steps 17-22, Exonuclease I treatment: 1.5 h

Steps 23-27, Preparation of splint oligos: 45 min

### Day 3

Steps 28-33, Ligation of splint oligos onto cDNA: 1.5 h

Steps 34-38, Proteinase K digestion: 20 min

Steps 39-51, AMPureXP cleanup: 1 h

Steps 52-56, Test amplification qPCR: 5.5 h

### Day 4

Steps 57-61, PCR: 5.5 h

Steps 62-72, AMPureXP cleanup: 1 h

Steps 73 and 74, Quantifying DNA length and concentration: 1 h

### Days 5-7

Step 75, ONT library preparation: 2 d

### Days 8-15

Steps 76-96, Data analysis: ~7 d

### Anticipated results

Step 73) After final PCR amplification it is possible to determine the quality of the cDNA per cell. This can be performed on an Agilent Bioanalyzer or an Agilent TapeStation. A good cDNA has an average size of 1.8-2.5 kb. If RNA degradation took place, small fragments will be found in the cDNA profile (Figure 6A), and if cell lysis was unsuccessful, no peak will be observed in the trace (Figure 6B). If a very sharp peak at the start of the trace is found at around 100bp, this can indicate the presence of primer dimers, and we encourage another AMPureXP bead cleanup. This is the library that will be sequenced and should consist of a clear peak at ~2 kb when ran on a TapeStation or Bioanalyzer (Figure 6C).

Step 77) Sequencing of 200 fmol of cDNA will yield around 10 million reads on a MinION or around 80 million reads on a PromethION device, respectively (Figure 7C). An equal distribution of forward and reverse reads is expected as the libraries are stranded. Reads should be assigned evenly across all ONT barcodes, and span transcript isoforms.

Step 79) Less than 20% of reads should contain internal adapters and less than 10% of reads lack both adapters at their ends. Internal_adaptor_QC.Rmd will output these statistics. Of all reads, 75% should remain after adapter trimming (Figure 7C).

Step 82) Demultiplexed reads should show high alignment scores (around 40) and a large gap (approximately 100) for alignment to each barcode (Figure 7D). Approximately 70% of reads should remain after demultiplexing (Figure 7C).

Step 88) 40-80 million reads from PromethION sequencing should result in around ~500,000 reads per cell and approximately 200-300 groups per barcode per cell. 25% of all reads should be error corrected to transcripts, depending on PCR duplication rate (Figure 7C).

Step 93) The flair output.gtf can be viewed on a genome browser to evaluate isoform assignment as well as read depth for genes and TEs (Figure 8A-D).

Step 96) The reads per TE family and number of TEs will depend on the sample. For example, 6 blastomeres of 2-cell stage embryos with 300,000 transcripts per cell show 5000 expressed genes and 15,000 expressed TEs per cell (Figure 8E,F). 96 human iPSCs show around 1000 expressed genes with around 500 TEs expressed per cell (Figure 8E,F). The mean expression in 2-cell stage embryos is 2.5 reads per million (RPM) compared to 0.25 RPM for human iPSCs (Figure 8F).

## Extended Data

## Supplementary Material

Supplementary Materials

## Figures and Tables

**Table 1:** Troubleshooting table

Step	Problem	Possible Reason	Possible Solution
73	Low cDNA library yield.	Too few PCR cycles.	Test cycle requirement prior to PCR using qPCR step.
	Low cDNA library yield.	Too few PCR cycles.	Amplify library with additional PCR cycles.
		Cells dead prior to collection.	Ensure cells kept in conditions close to those in the tissue/culture of origin prior to sorting. Check quality by staining for dead cells.
	cDNA profile is much shorter than expected.	RNA degradation prior to library preparation	Ensure cells are kept as close to physiological conditions prior to sorting. Ensure lysis buffer is present in each well and contains RNase inhibitor. Keep samples on ice prior to RT.
	Large peak of short fragments in library.	Incomplete removal of primers during AMPure clean-up.	Reduce number of PCR cycles or use a lower ratio of AMPure beads:cDNA to size select.
78	High number of reads with internal adaptors.	Read ligation during nanopore library prep.	Use split_reads.sh and porechop.img to split reads containing internal adaptors.
90	High numbers of PCR duplicates.	Overamplification of cDNA library.	Optimise cycles for PCR with qPCR step, reduce number of cycles.

## Data Availability

Example results were generated by analysing E-MTAB-9577 (ref. ^19^) https://www.ebi.ac.uk/biostudies/arrayexpress/studies/E-MTAB-9577. Test dataset of 1x10^5^ basecalled reads with 50 nt UMIs available at: https://github.com/Berrenslab/CELLOseq_50ntUMI/blob/main/example%20data/E14CELLO_plate_8_50nt_test.fastq.gz

## Key points

- CELLO-seq enables high-fidelity mapping of expression data from highly sequence-similar transposable elements (TEs) to unique genomic loci. This is through the use of long UMIs and a high number of PCR duplicates to correct errors from long read sequencing.
- Compared to other methods, CELLO-seq allows single-cell sequencing of the transcriptome to be combined with the ability to map locus-specific expression of TEs across all TE sub-families.

## Code availability

For data analysis the code is available in the following GitHub repositories: https://github.com/MarioniLab/CELLOseq, https://github.com/MarioniLab/sarlacc, https://github.com/MarioniLab/long_read_simulations.

50 nt UMI scripts and the full analysis pipeline used for this paper is available at: https://github.com/Berrenslab/CELLOseq_50ntUMI/.
